# Exosomes derived from bladder epithelial cells infected with uropathogenic *Escherichia coli* increase the severity of urinary tract infections (UTIs) by impairing macrophage function

**DOI:** 10.1371/journal.ppat.1011926

**Published:** 2024-01-08

**Authors:** Zihao Wang, Ziming Jiang, Yu Zhang, Congwei Wang, Zhaoyang Liu, Zhankui Jia, Sudhanshu Bhushan, Jinjian Yang, Zhengguo Zhang

**Affiliations:** 1 Department of Urology, The First Affiliated Hospital of Zhengzhou University, Zhengzhou, Henan, China; 2 Institute of Anatomy and Cell Biology, Unit of Reproductive Biology, Justus-Liebig-University Giessen, Giessen, Germany; University of Utah, UNITED STATES

## Abstract

Uropathogenic *Escherichia coli* (UPEC) is the primary causative agent of urinary tract infections (UTIs) in humans. Moreover, as one of the most common bacterial pathogens, UPEC imposes a substantial burden on healthcare systems worldwide. Epithelial cells and macrophages are two major components of the innate immune system, which play critical roles in defending the bladder against UPEC invasion. Yet, the routes of communication between these cells during UTI pathogenesis are still not fully understood. In the present study, we investigated the role of membrane-bound nanovesicles (exosomes) in the communication between bladder epithelial cells and macrophages during UPEC infection, using an array of techniques such as flow cytometry, miRNA profiling, RNA sequencing, and western blotting. Moreover, our in vitro findings were validated in a mouse model of UPEC-induced cystitis. We found that UPEC infection induced the bladder epithelial MB49 cell line to secrete large numbers of exosomes (MB49-U-Exo), which were efficiently absorbed by macrophages both in vivo and in vitro. Assimilation of MB49-U-Exo induced macrophages to produce proinflammatory cytokines, including tumor necrosis factor (TNF)α. Exposure of macrophages to MB49-U-Exo reduced their phagocytic activity (by downregulating the expression of phagocytosis-related genes) and increased their rate of apoptosis. Mechanistically, we showed that MB49-U-Exo were enriched in miR-18a-5p, which induced TNFα expression in macrophages by targeting PTEN and activating the MAPK/JNK signaling pathway. Moreover, administration of the exosome secretion inhibitor GW4869 or a TNFα-neutralizing antibody alleviated UPEC-mediated tissue damage in mice with UPEC-induced cystitis by reducing the bacterial burden of the bladder and dampening the associated inflammatory response. Collectively, these findings suggest that MB49-U-Exo regulate macrophage function in a way that exacerbates UPEC-mediated tissue impairment. Thus, targeting exosomal -release or TNFα signaling during UPEC infection may represent promising non-antibiotic strategies for treating UTIs.

## Author summary

Urinary tract infections, which are typically caused by uropathogenic *Escherichia coli* (UPEC), affect over 150 million people each year, worldwide. Because the incidence of antibiotic resistance among UPEC strains is on the rise, urinary tract infections are becoming increasingly challenging to treat using this standard treatment method. Moreover, although the host immune response against UPEC has been intensively studied, the mechanism of communication between bladder epithelial cells and macrophages during UPEC infection remains largely unexplored. Here, we characterized the exosomes released from bladder epithelial cells following UPEC infection and evaluated their impact on macrophages. We demonstrated that bladder tissue destruction following excessive innate immune cell activation during UPEC infection may be driven by exosomes secreted from bladder epithelial cells. We further showed that pharmacologically inhibiting exosomal release from epithelial cells limited macrophage-associated inflammation, innate immune cell infiltration, and histopathological injuries in a mouse model of UPEC-induced cystitis. Collectively, our findings suggest that modulating exosome release represents a promising non-antibiotic therapy for UTIs.

## Introduction

Urinary tract infections (UTIs), predominantly caused by uropathogenic *Escherichia coli* (UPEC), pose a serious health problem and major economic burden worldwide [1,2]. The increasing rate of bacterial resistance to antibiotics and the high recurrence rate of UPEC infections complicates UTI treatment. Thus, a better understanding of the pathogenesis of UPEC infections and the related host immune response is urgently needed to develop novel therapeutic strategies that do not rely on antibiotics [3–6].

The innate immune system of the bladder comprises epithelial cells and various resident or infiltrating immune cells. Together, these components recognize pathogens and induce proinflammatory immune responses via their pattern recognition receptors (e.g., Toll-like receptor 4, 7]. However, the excessive inflammation caused by an overactive immune response can exacerbate UTI symptoms and promote tissue destruction. As the first line of defense, the epithelial cells lining the bladder prevent UPEC infection by secreting soluble antimicrobial agents or proinflammatory cytokines, which further recruit immune cells to eradicate the bacteria. Reportedly, UPEC induces the exfoliation of the bladder urothelium [8], which causes the clearance of infected cells and temporarily decreases the bacterial burden in the bladder. However, the process of exfoliation also leaves the underlying urethelial layer vulnerable to UPEC reinfection. As innate immune sentinels, macrophages play an essential role in defending the host against invading pathogens such as UPEC. Macrophages detect both pathogen- and danger-associated molecular patterns to initiate proinflammatory immune responses and coordinate neutrophil recruitment [9]. The majority of studies investigating the host immune response to UPEC infection have either focused on epithelial cells or macrophages in isolation [10–13]; however, few studies have addressed the importance of the crosstalk between these cells in UTI pathogenesis. Evidence from a murine model of UPEC-induced cystitis suggests that two subsets of macrophages (resident Ly6C^−^macrophages and recruited Ly6C^+^macrophages) work in concert to coordinate the recruitment of neutrophils to participate in antimicrobial defense [14]; however, how bladder epithelial cells, as the primary barrier in the urinary tract and the first responders to pathogens, communicate with macrophages during UPEC infection is still not fully understood.

Exosomes are membrane-derived vesicles secreted by all cell types, which are key mediators of cell-cell communication [15,16]. Emerging evidence indicates that exosomes play essential roles in the regulation of host immune responses during pathogenic infection [17,18]. For instance, exosomes derived from virally-infected cells induce the apoptosis of T cells and monocytes via ADP-ribose-polymerase-1- and caspase-3-dependent mechanisms to inhibit the immune response and facilitate viral spread [19]. Moreover, exosomal miRNA secreted by macrophages infected with *Helicobacter pylori* was shown to regulate the immune response by promoting the production of cytokines such as tumor necrosis factor (TNF)α, interleukin (IL)-6, and IL-23[20]. Meanwhile, exosomes derived from intestinal epithelial cells infected with adherent invasive *E*. *coli* (AIEC) can transfer specific miRNAs to recipient intestinal epithelial cells to suppress the autophagy-mediated clearance of intracellular AIEC [21]. Therefore, exosomes may act as intermediaries to coordinate epithelial and immune cell functions during UPEC infection.

The aim of the present study was to investigate the role of exosomes in the communication between epithelial cells and macrophages during UPEC infection. We began by characterizing the exosomes released from bladder epithelial cells following UPEC infection. We then explored the impact of these exosomes on macrophages in vitro and in vivo. Finally, we investigated the influence of exosomes on bladder histopathological outcomes by pharmacologically targeting exosome release in a murine model of UPEC-induced cystitis.

## Results

### Macrophages assimilate exosomes secreted by bladder epithelial cells both in vitro and in vivo

**Fig 1:**
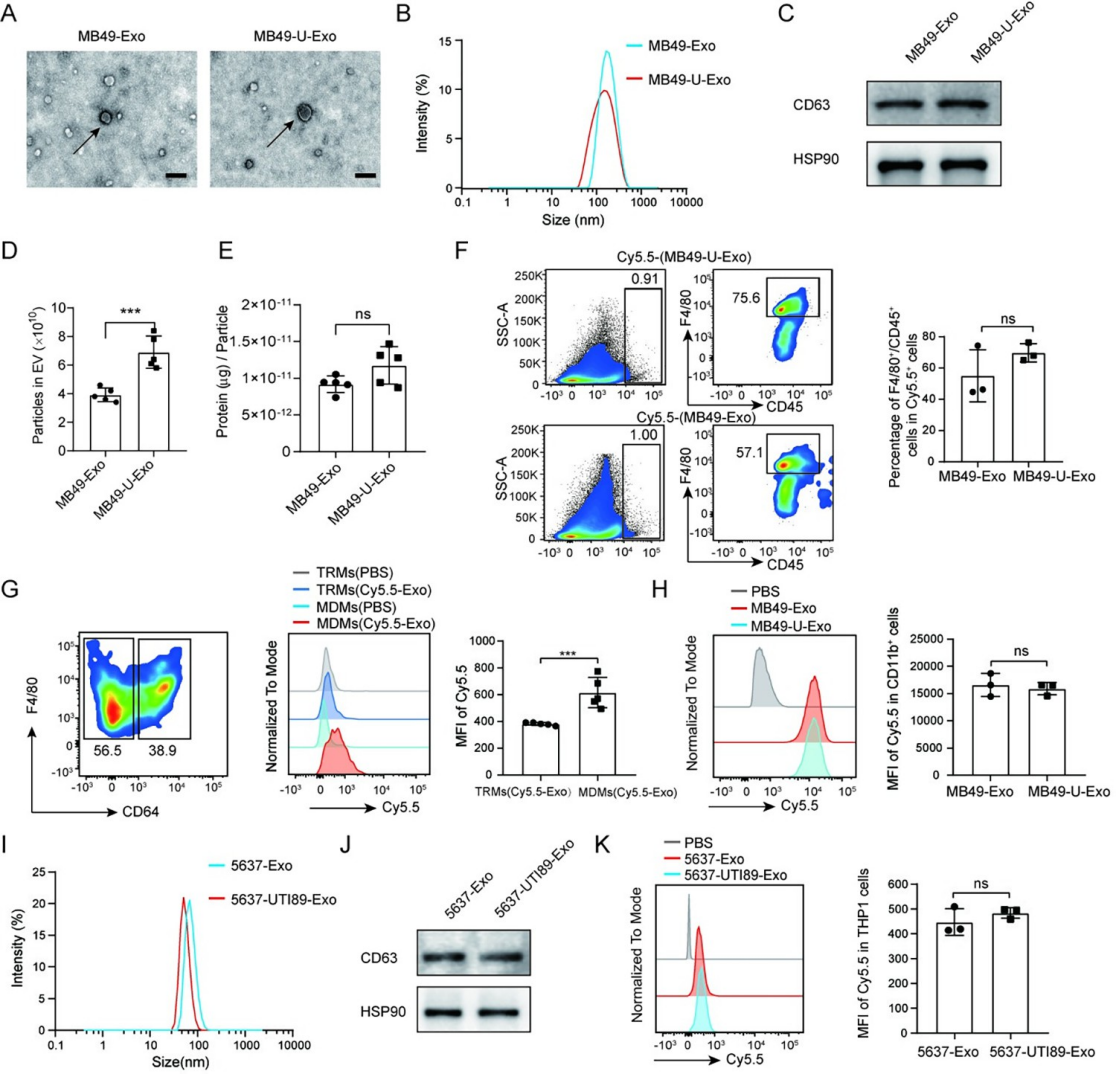
Macrophages assimilate exosomes secreted by bladder epithelial cells both in vitro and in vivo. (A) Representative transmission electron microscopy (TEM) images of exosomes from MB49 cells (MB49-Exo) and UPEC-infected MB49 cells (MB49-U-Exo). Scale bar = 100 nm. (B) The size distribution of exosomes was analyzed using a Malvern Zetasizer instrument. (C) Representative western blots showing the expression of CD63 and HSP90 in MB49-derived exosomes. (D) The total particle number in MB49-Exo and MB49-U-Exo isolated from the same volume of culture medium was determined using nanoparticle tracking analysis (n = 5; ***P < 0.001; Student’s t-test). (E) The BCA assay was used to determine the protein content in exosomes isolated form different treatment conditions (n = 5; ns: no significance; Student’s t-test). (F) Cy5.5-labeled MB49-U-Exo/MB49-Exo or unlabeled exosomes (PBS control) were injected to mouse bladders; the percentage of Cy5.5^+^ cells in bladder 12 h post-injection was quantified by flow cytometry. Representative images showing the gating strategy for analyzing Cy5.5^+^ cells (left) and bar graphs showing the CD45^+^F4/80^+^ macrophage frequency among the Cy5.5^+^ cells (right) (n = 3, ns: no significance; Student’s t-test). (G) Histograms and bar graphs showing the Cy5.5 mean fluorescence intensity (MFI) of the CD64^+^ tissue-resident macrophages (TRMs) and CD64^−^ monocyte-derived macrophages (MDMs) (n = 5; ***P < 0.001; Student’s t-test). (H) Cy5.5 MFI of BMDMs after treatment with Cy5.5-labeled exosomes or PBS (control) at the indicated concentration for 3 h. Histograms and bar graphs showing MFI of macrophages after exosome absorption (n = 3; ns: no significance; Student’s t-test). (I) Size distribution of exosomes from uninfected (5637-Exo) and UTI89-infected (5637-UTI89-Exo) 5637 cells. (J) Representative western blots showing the expression of CD63 and HSP90 in 5637 exosomes. (K) Flow cytometric analysis of Cy5.5 MFI in THP1 cells after treatment with Cy5.5-labeled exosomes or PBS (control) for 3 h. Histograms and bar graphs showing Cy5.5 MFI of THP1 cells after exosome absorption (n = 3; ns: no significance; Student’s t-test).

Exosomes play important roles in intercellular communication. Hence, to explore the interactions between bladder epithelial cells and immune cells during UPEC infection, we isolated exosomes from the culture medium of the murine bladder epithelial cell line MB49, cultured in the presence or absence of UPEC strain 536. The typical cup-shaped morphology of exosomes derived from uninfected (MB49-Exo) or UPEC-infected (MB49-U-Exo) MB49 cells was confirmed using transmission electron microscopy (Fig 1A). Moreover, particle size analysis and western blotting were used to assess the exosomal size distribution and the protein expression of the exosomal markers CD63 and HSP90, respectively (Fig 1B and 1C). Intriguingly, the number of exosomes derived from MB49 cells was significantly increased following UPEC infection (Fig 1D and 1E). To track the cellular absorption of exosomes secreted from MB49 cells in vivo, we labelled exosomes with Cy5.5 and injected them into the bladder of a female mouse. Flow cytometry analysis revealed that the majority of both MB49-Exo and MB49-U-Exo were taken up by CD45^+^F4/80^+^ bladder macrophages (Fig 1F). Moreover, the CD64^−^F4/80^+^, monocyte-derived macrophages (MDMs) took up more of the Cy5.5-labeled MB49-U-Exo than the CD64^+^F4/80^+^, tissue-resident macrophages (TRMs) (Fig 1G). Similarly, the flow cytometric and immunofluorescence analyses revealed that the bone-marrow-derived macrophages (BMDMs) absorbed both Cy5.5-labeled MB49-Exo and MB49-U-Exo (Figs 1H and S1A). We next verified our results by using the human bladder epithelial cell line 5637 and another UPEC strain (UTI89). We did not observe any difference between these UPEC stains in terms of their adhesion and invasion of 5637 and MB49 cells (S1B and S1C Fig). Moreover, the results of the lactate dehydrogenase activity assay showed that neither strain caused 5637 or MB49 cell death after a 24-hour infection period (S1D Fig). As expected, both the UTI89 and 536 UPEC strains induced 5637 cells to secrete exosomes; which was verified by analyzing particle size distribution and the expression of CD63 and HSP90 (Figs 1I, 1J, S1E, and S1F). We next used flow cytometry to show that the human monocytic cells line THP1 assimilated Cy5.5-labeled 5637-Exo and 5637-UTI89-Exo (Fig 1K), as well as 5637-Exo and 5637-UPEC536-Exo (S1G Fig). Collectively, these findings suggest that UPEC treatment significantly increased the release of exosomes from bladder epithelial cells, and—these exosomes were taken up by macrophages both in vitro and in vivo.

### MB49-U-Exo exacerbate UPEC-induced cystitis

**Fig 2:**
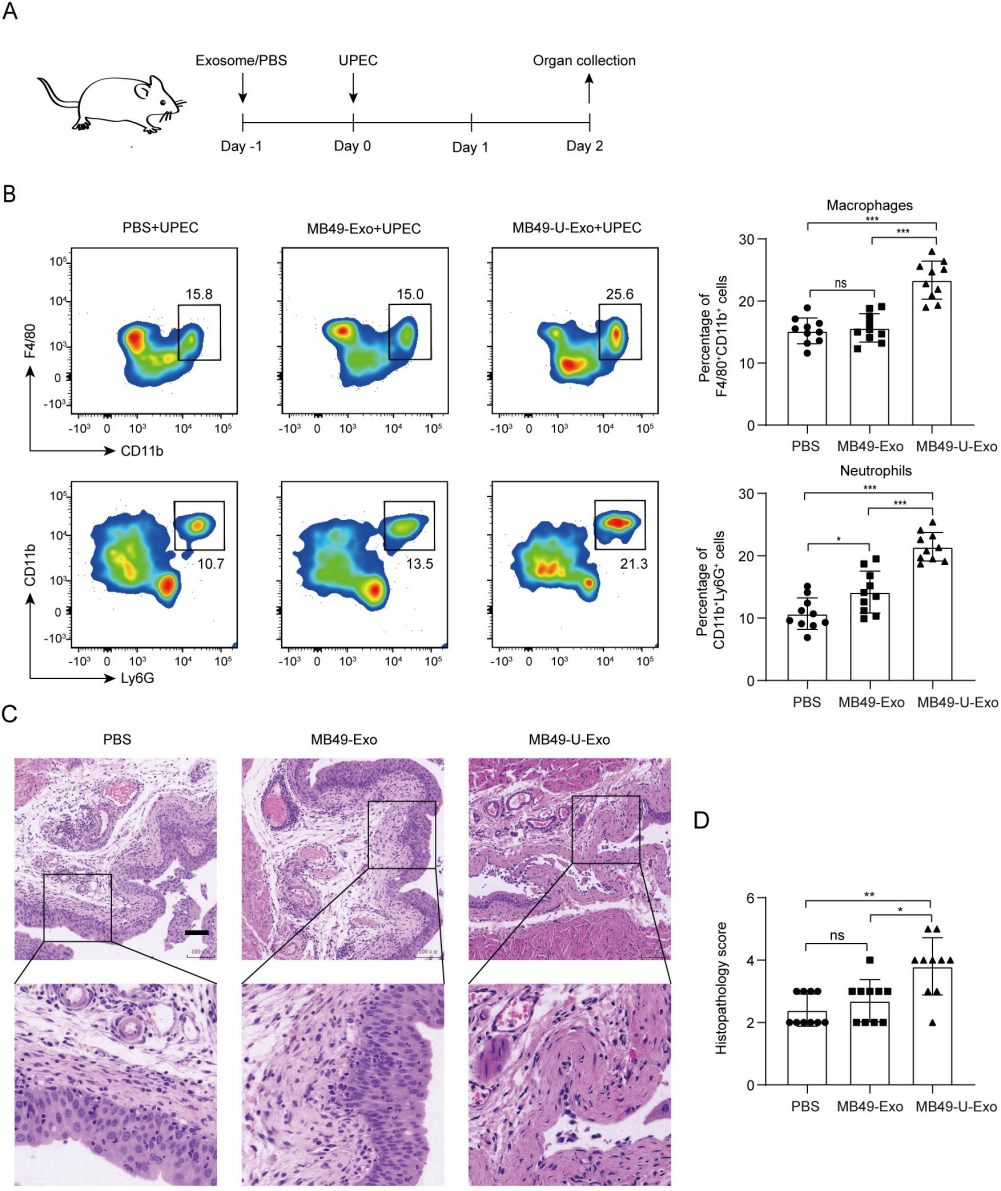
MB49-U-Exo exacerbate the histopathology of UPEC-induced cystitis. (A) Schematic diagram outlining the MB49-Exo or MB49-U-Exo pretreatment of mice with UPEC-induced cystitis. Exosomes (1 μg in 100 μL PBS) were injected into the mouse bladder via the urethra; 1 × 10^8^ CFU UPEC were inoculated into the bladder (via the urethra) 1 day later. (B) Flow cytometric analysis of macrophage and neutrophil frequencies in the bladder at 2 days post-infection. Images and bar graphs showing populations of F4/80^+^CD11b^+^ macrophages and CD11b^+^Ly6G^+^ neutrophils in each group (data from 3–4 animals per group, and three independent experiments are shown; ns: no significance, *P < 0.05, ***P < 0.001; one-way ANOVA). (C) Representative images of hematoxylin- and eosin-stained bladder sections from the indicated treatment groups. (D) Bar graphs comparing the group histopathology scores (data from 3–4 animals per group and three independent experiments are shown; ns: no significance, *P < 0.05, **P < 0.01; one-way ANOVA).

We next evaluated the potential effects of MB49-U-Exo on the pathogenesis of UPEC-induced cystitis. To this end, we established a mouse model with UPEC-induced cystitis, which we pretreated with MB49-Exo or MB49-U-Exo (Fig 2A). The results of the flow cytometric analysis revealed that the number of F4/80^+^CD11b^+^ macrophages and CD11b^+^Ly6G^+^ neutrophils in the MB49-U-Exo group was significantly higher at 2 days post-UPEC infection than in the MB49-Exo group (Fig 2B). Moreover, pretreating mice with MB49-U-Exo exacerbated their cystitis pathology, as evidenced by the higher percentages of infiltrating epithelial and subepithelial inflammatory cells (Fig 2B and 2C) and the significant increase in their histopathological score in comparison with mice pretreated with MB49-Exo or PBS (Fig 2D). These data suggest that MB49-U-Exo treatment augmented the innate inflammatory response against UPEC infection, resulting in tissue destruction.

### MB49-U-Exo induce macrophages to secrete proinflammatory cytokines

**Fig 3:**
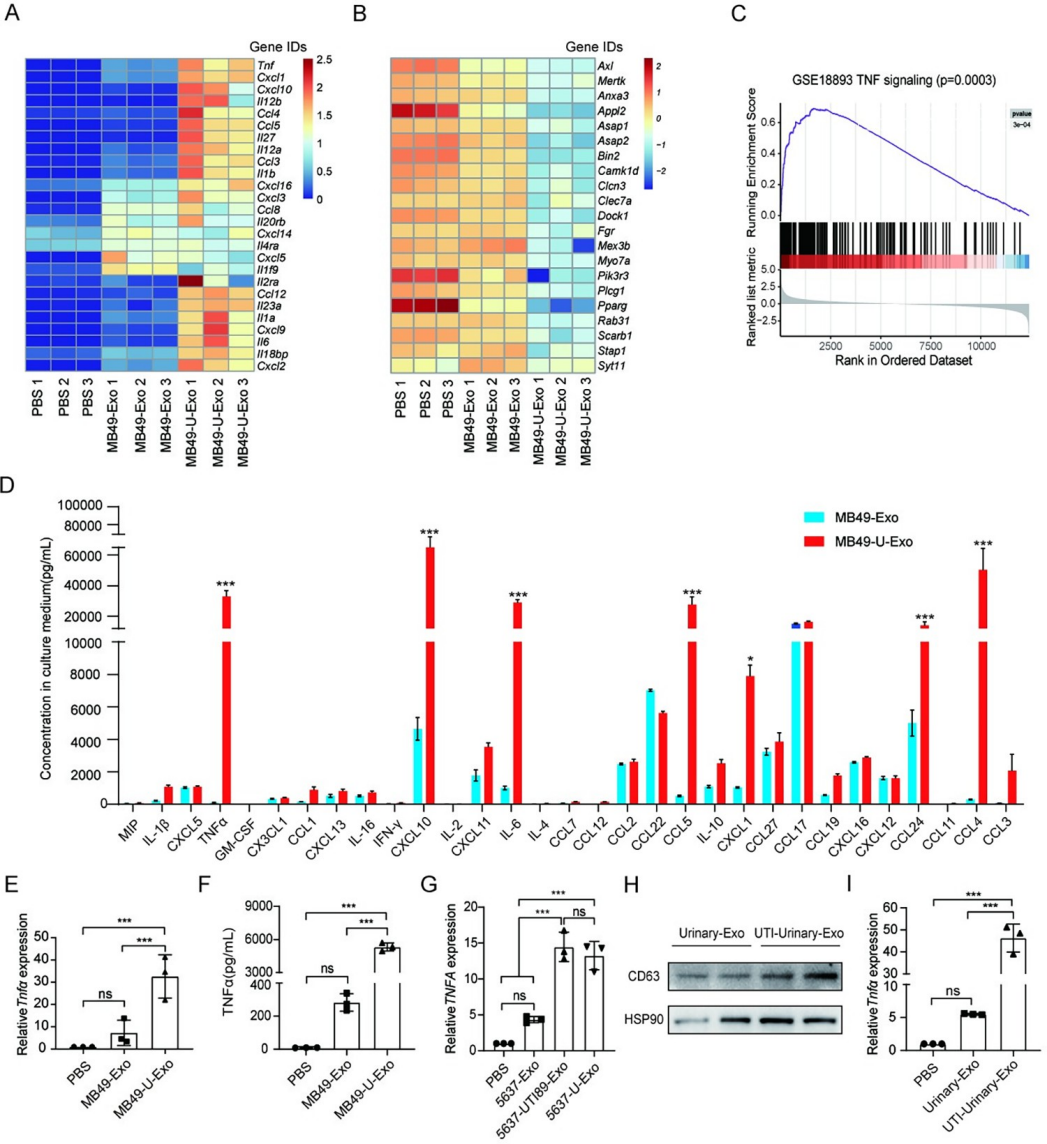
MB49-U-Exo induce macrophages to secrete proinflammatory cytokines. (A-C) BMDMs were stimulated with MB49-Exo or MB49-U-Exo for 3 h; their gene expression profiles were then evaluated by RNA sequencing analysis. (A) Heat map of differentially expressed inflammatory genes in BMDMs following PBS or exosome treatment (n = 3 per sample). (B) Heat map showing the differential expression of phagocytosis-related genes. (C) Results of GSEA, showing the enrichments of upregulated genes associated with TNF signaling in the MB49-U-Exo- vs. MB49-Exo-treated BMDMs. (D) Cytokine array of BMDMs after exosome stimulation (n = 3; *P < 0.05, ***P < 0.001; two-way ANOVA). (E) qRT-PCR analysis of *Tnfα* expression levels in exosome-treated BMDMs (n = 3; ns: no significance, ***P < 0.001; one-way ANOVA). (F) Concentration of TNFα in the conditioned medium of PBS- (control) or exosome-treated BMDMs, measured by ELISA (n = 3; ns: no significance, ***P < 0.001; one-way ANOVA). (G) qRT-PCR analysis of *TNFA* expression levels in exosome-treated THP1 cells (n = 3; ns: no significance, ***P < 0.001; one-way ANOVA). (H-I) A model of UPEC-induced cystitis was established by injecting 1 × 10^8^ CFU UPEC into the mouse bladder via the urethra. Urine was collected and pooled from three animals 1 day post-infection. The exosomes were extracted from the urine by ultracentrifugation. Data are presented from three independent experiments. (H) Representative western blots showing the expression of exosome markers CD63 and HSP90 in the urine-derived exosomes. (I) qRT-PCR analysis of *Tnfα* expression levels in BMDMs treated with urine-derived exosomes (ns: no significance, ***P < 0.001; one-way ANOVA).

To further explore the molecular mechanisms underlying the exacerbation of UPEC-induced cystitis by MB49-U-Exo administration, we treated macrophages with MB49-U-Exo. We excluded the possibility of lipopolysaccharide (LPS) contamination during the exosome isolation procedure by measuring the bacterial endotoxin activity, and confirmed that both MB49-U-Exo and MB49-Exo had low endotoxin activity (S2A Fig). We next performed an RNA sequencing analysis of BMDMs exposed to MB49-U-Exo or MB49-Exo and identified 1,311 differentially expressed genes (DEGs; 828 upregulated and 483 downregulated) in MB49-U-Exo-treated vs. MB49-Exo-treated BMDMs (S2B Fig). A Kyoto Encyclopedia of Genes and Genomes (KEGG) analysis was then performed to show that the upregulated DEGs were implicated in pathways related to infectious disease and the immune system (S2C Fig). Among the DEGs, multiple inflammatory genes (e.g., *Tnf*, *Il6*, *Cxcl10*, and *Ccl4*) were markedly upregulated (Fig 3A), whereas a variety of genes related to phagocytosis (e.g., *Axl*, *Mertk*, *Appl2*, *Clec7a*) were significantly downregulated in MB49-U-Exo- vs. MB49-Exo-treated BMDMs (Fig 3B). Gene set enrichment analysis (GSEA) confirmed that MB49-U-Exo treatment upregulated genes involved in TNFα signaling in BMDMs (Fig 3C). In accordance, a cytokine array analysis confirmed that MB49-U-Exo promoted the secretion of inflammatory cytokines and chemokines (e.g., TNFα, CXCL10, IL-6, CCL5, CXCL1, CCL24, and CCL4) by macrophages (Fig 3D). Among these, the potent proinflammatory cytokine TNFα has been shown to be a critical regulator of innate immunity against UPEC infection in the bladder [14]. Indeed, we found that the expression of TNFα, at both the mRNA and protein levels, was markedly higher in BMDMs treated with MB49-U-Exo than in those treated with MB49-Exo or PBS (Fig 3E and 3F). Similarly, exosomes derived from 5637 cells infected with the 536 or UTI89 UPEC strains also induced TNFα expression in THP1 cells (Fig 3G). We next asked whether exosomes isolated from the urine of mice with UPEC-induced cystitis could regulate TNFα expression in macrophages. Again, detection of CD63 and HSP90 by western blotting was used to confirm the successful isolation of exosomes from urine (Fig 3H). As expected, the expression of TNFα was significantly higher in BMDMs treated with exosomes isolated from UPEC-infected mouse urine than in those treated with exosomes from the urine of PBS-treated animals or PBS treatment (Fig 3I).

### MB49-U-Exo induce TNFα production in macrophages by activating the MAPK/ERK/JNK pathway

**Fig 4:**
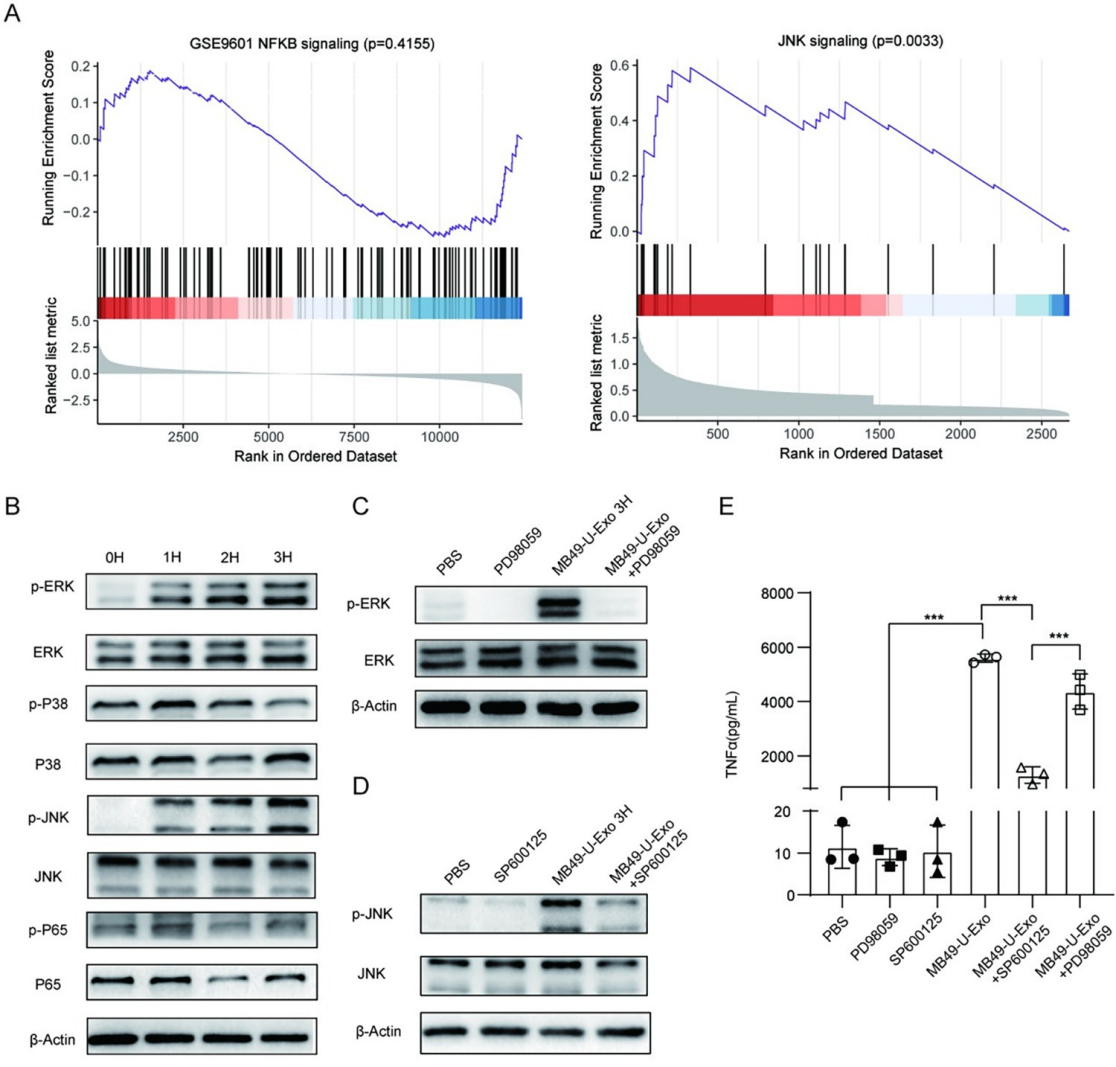
MB49-U-Exo induce TNFα production in macrophages via the activation of the MAPK/ERK/JNK pathway. (A) Results of the GSEA revealed that MB49-U-Exo-treated BMDMs were enriched in upregulated genes associated with JNK signaling but not NF-κB signaling, compared with MB49-Exo-treated BMDMs. (B) Western blot showing the expression of phosphorylated and total ERK, P38, JNK, and P65 in BMDMs treated with MB49-U-Exo for the indicated time periods. (C) Expression of p-ERK/ERK in BMDMs stimulated with either PBS, PD98059 (20 μM), MB49-U-Exo, or a combination of MB49-U-Exo and PD98059 for 3 h. (D) Western blot showing the expression of p-JNK/JNK in BMDMs stimulated with either PBS, SP600125 (40 nM), MB49-U-Exo, or a combination of MB49-U-Exo and SP600125 for 3 h. β-actin was used as a loading control. Data are presented from three independent experiments. (E) Production of TNFα by BMDMs after PBS, MB49-U-Exo, and/or PD98059 and SP600125 treatment, determined by ELISA. (n = 3, ***P < 0.001; one-way ANOVA).

Having shown that MB49-U-Exo administration increased the production of a variety of inflammatory cytokines and chemokines by BMDMs, we next sought to determine which signaling pathways were involved in this process. GSEA of genes enriched in MB49-U-Exo-treated vs. MB49-Exo-treated BMDMs suggested the c-Jun N-terminal kinase (JNK) rather than the nuclear factor kappa light chain enhancer of activated B cells (NF-κB) signaling pathway was implicated (Fig 4A). Hence, the phosphorylated and total protein expression levels of extracellular signal-regulated kinase (ERK), P38, JNK, and P65 were next determined by western blotting. Interestingly, the levels of phosphorylated (p)-ERK and p-JNK were markedly increased following MB49-U-Exo, suggesting that MB49-U-Exo caused MAPK/ERK/JNK signaling pathway activation (Figs 4B and S3A). Meanwhile, MB49-Exo treatment activated the ERK but not JNK branch of the MAPK signaling pathway (S4 Fig). To further identify the effect of ERK/JNK activation on TNFα expression, we pretreated the BMDMs with the ERK inhibitor PD98059 or the JNK inhibitor SP600125. We found that PD98059 treatment significantly reduced MB49-U-Exo-induced ERK activation as well as TNFα secretion (Figs 4C, 4E, and S3B); meanwhile, SP600125 treatment had an even more potent inhibitory effect on TNFα production (Figs 4D, 4E, and S3C). These findings show that MB49-U-Exo induced TNFα production in BMDMs by activating the MAPK/ERK/JNK pathway.

### MB49-U-Exo trigger macrophage apoptosis via the autocrine production of TNFα

**Fig 5:**
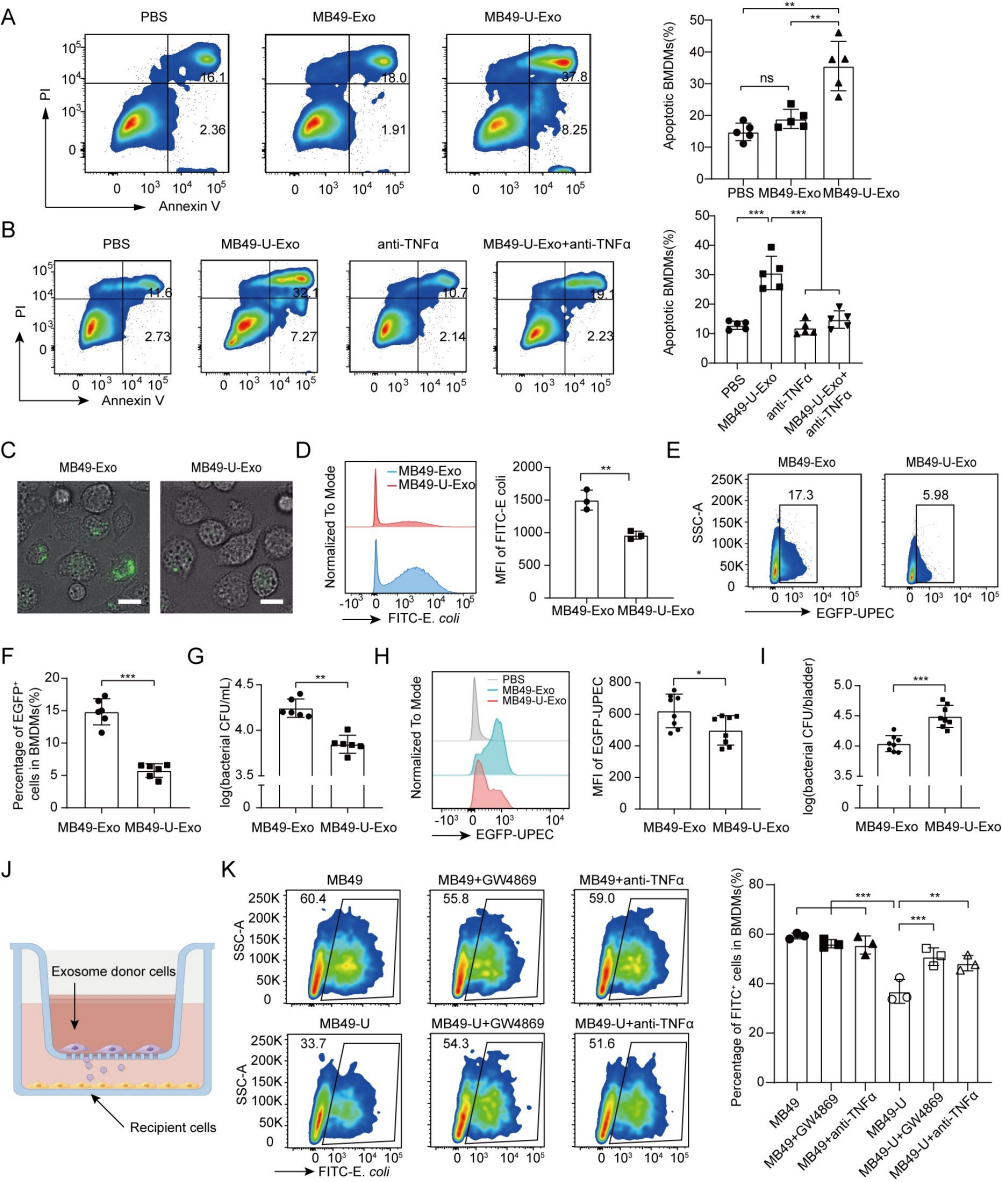
MB49-U-Exo trigger macrophage apoptosis, via the autocrine production of TNFα, and attenuate macrophage phagocytic capacity. (A) Flow cytometric analysis of BMDMs treated with PBS, MB49-Exo, or MB49-U-Exo and stained for Annexin V and with PI (n = 5; **P < 0.01; one-way ANOVA). (B) The percentage of apoptotic BMDMs after exposure to PBS, MB49-U-Exo, a TNFα-neutralizing antibody (100 ng/mL), or a combination of these reagents, was determined by flow cytometry (n = 5; ***P < 0.001; one-way ANOVA). (C-D) BMDMs were treated with MB49-Exo/MB49-U-Exo (10 μg/mL) for 24 h and then with FITC-labelled *E*. *coli* BioParticles (50 μg/mL) for 1 h, and phagocytosis activity was determined using confocal microscopy (scale bar = 20 μm) and flow cytometry. Representative confocal microscopy images of the FITC signal (C) and flow cytometry histograms of the FITC MFI (D) of BMDMs are shown (n = 3; **P < 0.01; Student’s t-test). (E-G) Phagocytic activity of BMDMs pretreated with the indicated types of exosomes and cocultured with EGFP-expressing *E*. *coli* (green), was determined by flow cytometry and the CFU counting assay. (E-F) Images and bar graphs showing the EGFP-positive cell populations in the two groups. (G) Bacterial burden was expressed in logCFU/mL (n = 6, **P < 0.01; ***P <0.001; Student’s t-test and Mann–Whitney *U* test). (H-I) C57BL/6J female mice were pretreatment with MB49-Exo or MB49-U-Exo and then infected with EGFP-UPEC. (H) Flow cytometric analysis of the EGFP MFI of F4/80^+^CD11b^+^ macrophages at 2 days post-infection. (I) Bladder bacterial burden was expressed in logCFU/bladder. (data are presented from 2–3 animals per group and from three independent experiments; *P < 0.05; ***P <0.001; Student’s t-test and Mann–Whitney *U* test). (J) Schematic diagram showing BMDMs (as exosome recipients) in the bottom well of the Transwell system and UPEC-infected or uninfected MB49 cells (as exosome donors) in the upper insert. Cells were treated with GW4869 (10 μg/mL) or a TNFα-neutralizing antibody (100 ng/mL) for 12 h, or left untreated. (K) The cocultured BMDMs were incubated with FITC-labelled *E*. *coli* BioParticles (50 μg/mL) for 1 h, before their phagocytic activity was assessed by flow cytometry. Plots show the flow cytometry gating strategy. Bar graphs show FITC-positive cell populations in each group (n = 3, **P < 0.01, ***P < 0.001; one-way ANOVA).

The fact that TNFα plays key roles in apoptosis, inflammation, and immunity[22], prompted us to investigate whether MB49-U-Exo treatment also induced macrophage apoptosis. Indeed, we observed significantly higher rates of apoptosis in BMDMs treated with MB49-U-Exo than in those treated with MB49-Exo or PBS (Fig 5A). In addition, using a TNFα-neutralizing antibody significantly decreased the extent of MB49-U-Exo-induced apoptosis (Fig 5B), suggesting that TNFα plays a proapoptotic role following macrophage exposure to MB49-U-Exo.

### MB49-U-Exo suppress macrophage phagocytic activity

Having determined that MB49-U-Exo induce the apoptosis of BMDMs and suppress the expression of phagocytosis-related genes, we next investigated their effect on the phagocytic activity of macrophages. MB49-U-Exo pretreatment significantly attenuated the phagocytic activity of BMDMs in comparison with MB49-Exo pretreatment, in a phagocytosis assay using FITC-labelled *E*. *coli* BioParticles (Fig 5C and 5D). Similarly, the ability of BMDMs to phagocytose live, EGFP-labeled UPEC was reduced following MB49-U-Exo but not MB49-Exo exposure (Fig 5E–5G). Furthermore, in the mouse model of UPEC-induced cystitis, MB49-U-Exo-pretreated bladder F4/80^+^CD11b^+^ macrophages engulfed fewer EGFP-UPEC than those pretreated with MB49-Exo (Fig 5H). As a result, the UPEC titer in the bladders of mice in the MB49-U-Exo group was much higher than that of mice in the MB49-Exo group, indicating that MB49-U-Exo attenuated the phagocytic capacity of macrophages in vivo (Fig 5I). To further assess the influence of exosomes on macrophage phagocytosis, we cocultured BMDMs and MB49 cells in a Transwell chamber, where the cells were separated by a 400-nm filter which only allowed the passage of exosomes (Fig 5J). The exosome-secretion inhibitor GW4869 or a TNFα-neutralizing antibody partially restored the ability of MB49-U-Exo-treated BMDMs to phagocytose FITC-labelled *E*. *coli* BioParticles (Fig 5K). Similar results were obtained using THP1 cells treated with 5637-U-Exo or 5637-Exo in combination with GW4869 or the TNFα-neutralizing antibody infliximab (S5 Fig).

### Inhibition of MB49-U-Exo-mediated TNFα production alleviates UPEC-elicited cystitis in vivo

**Fig 6:**
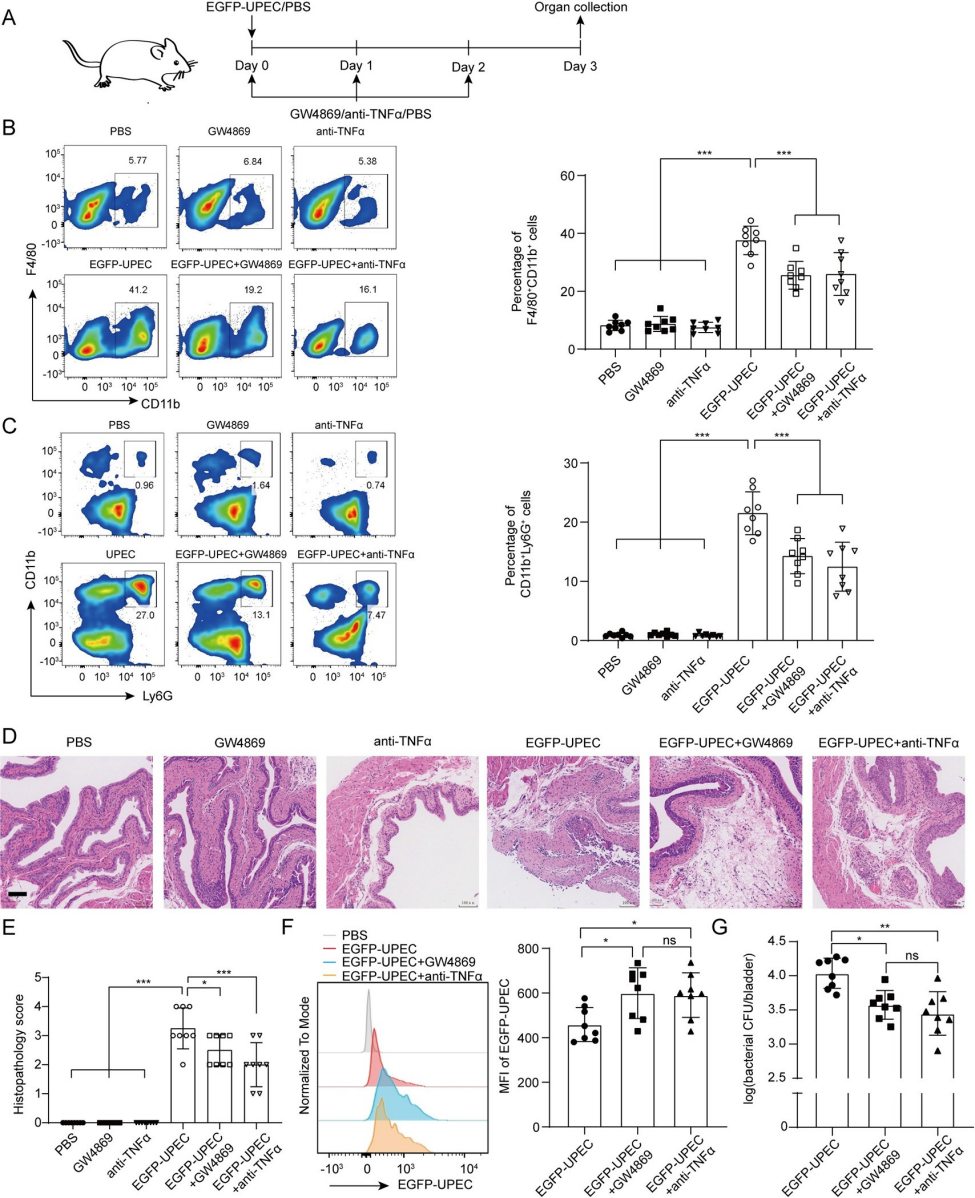
Inhibition of MB49-U-Exo-mediated TNFα production reduces the severity of UPEC-induced cystitis in vivo. (A) Schematic diagram outlining the treatment schedule. Mice were first infected with EGFP-UPEC to establish cystitis and then treated with either PBS, GW4869 (2.5 μg/g), a TNFα-neutralizing antibody (200 μg/kg), or a combination once daily. (B-C) Flow cytometric analysis of the frequency of F4/80^+^CD11b^+^ macrophages (B) and CD11b^+^Ly6G^+^ neutrophils (C) in the mouse bladder; bar graphs and representative images are shown (***P < 0.001; one-way ANOVA). (D) Representative images of H&E-stained bladder sections from the indicated treatment groups. (E) Comparison of group histopathology scores (data are presented as mean ± SD. *P < 0.05, ***P < 0.001; one-way ANOVA). (F) Flow cytometric analysis of the EGFP MFI of F4/80^+^CD11b^+^ macrophages after 3 days of infection. Histogram and bar graphs showing the EGFP MFI of macrophages after they have phagocytosed fluorescently-labelled bacteria (ns: no significance, *P < 0.05; one-way ANOVA). (G) Bladder bacterial counts of the three treatment groups expressed as logCFU/bladder (ns: no significance, *P < 0.05, **P < 0.01; Kruskal–Wallis test). Data are presented from 2–3 animals per group and from three independent experiments (B, C, and E-G).

As MB49-U-Exo administration induced macrophage apoptosis and suppressed their phagocytic activity, we next asked whether blocking exosome secretion or neutralizing TNFα activity could improve the severity of UPEC-induced cystitis. Indeed, the numbers of macrophages and neutrophils were significantly lower in the bladders of model mice with UPEC-induced cystitis (Fig 6A) that were treated with GW4869 or the TNFα-neutralizing antibody than in those that were not (Figs 6B, 6C, and S6A). Moreover, the histopathological scores of the GW4869 and TNFα-neutralizing antibody groups were significantly lower than that of the UPEC only group, as evidenced by the lower number of infiltrating epithelial/subepithelial inflammatory cells and the restoration of the surface epithelium (Fig 6B–6E). In addition to restoring the phagocytic capacity of BMDMs, we showed that GW4869 or TNFα-neutralizing antibody administration increased bacterial uptake by macrophages (Fig 6F). Consequently, the UPEC titers were significantly lower in the bladders of mice in the GW4869 and TNFα-neutralizing antibody treatment groups than in those of mice in the UPEC only group (Figs 6G and S6B). These findings suggest that blocking UPEC-mediated exosome secretion or TNFα production facilitated UPEC eradication by restoring macrophage phagocytic activity.

### MB49-U-Exo induce JNK activation and TNFα production by delivering miR-18a-5p

**Fig 7:**
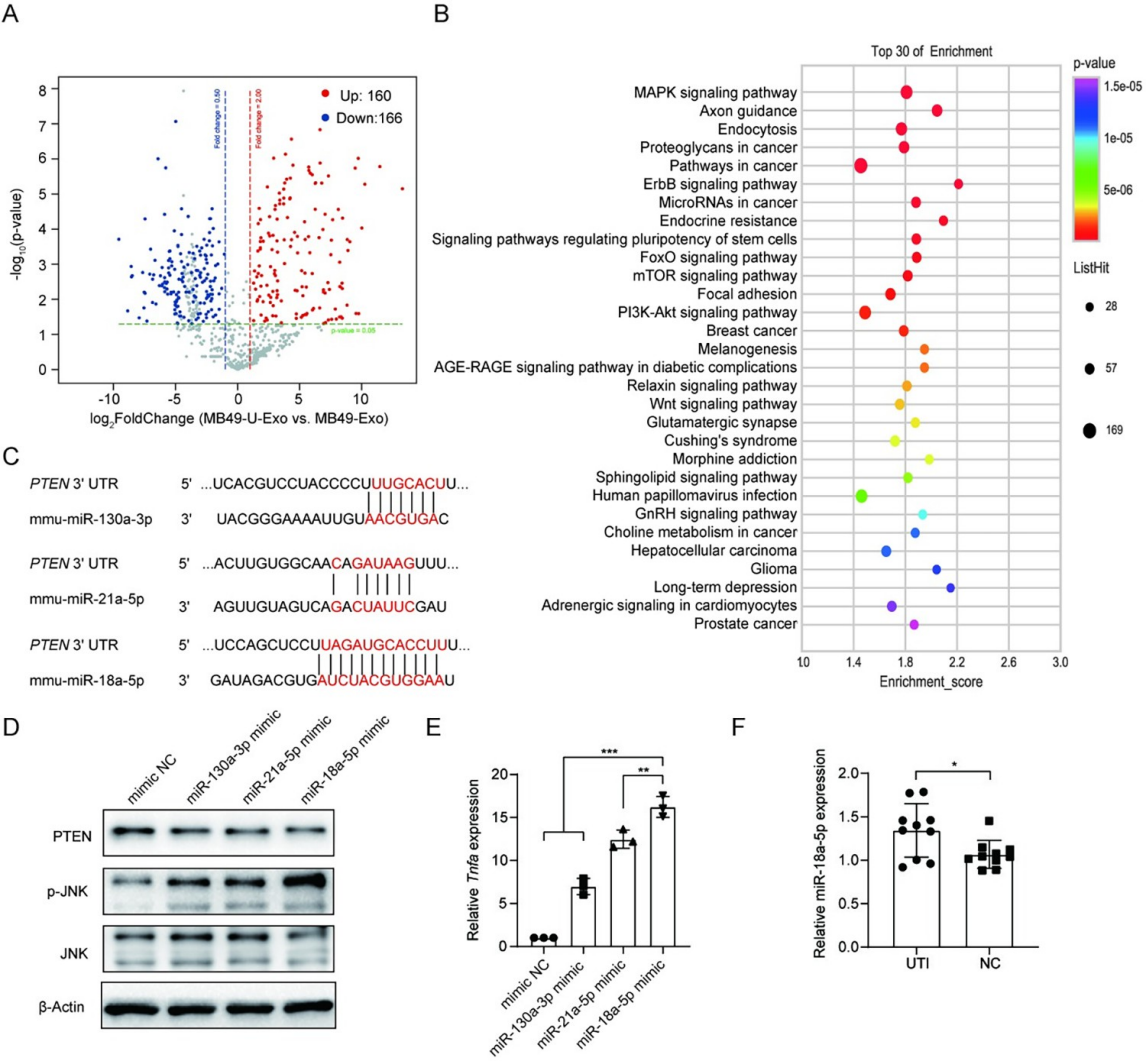
MB49-U-Exo induced JNK activation and TNFα expression in macrophages by delivering miR-18a-5p. (A) Volcano plot showing upregulated (red) and downregulated miRNAs (blue) in MB49-U-Exo vs. MB49-Exo, detected by microarray analysis. (B) Bubble plot of GO enrichment analysis results for MB49-Exo/MB49-U-Exo miRNA target genes. (C) Predicted binding sites for miR-130a-3p, miR-21a-5p, and miR-18a-5p within the PTEN 3’UTR. Seed sequences are marked in red. (D) Representative images of a western blot showing the expression of PTEN, p-JNK, and JNK in BMDMs 6 h after they were transfected with each miRNA mimic. Data are presented from three independent experiments. (E) qRT-PCR analysis of *Tnfα* expression levels in BMDMs treated with each miRNA mimic (n = 3, **P < 0.01, ***P < 0.001; one-way ANOVA). (F) Exosomes were isolated from the urine of patients with urinary tract infections and healthy volunteers (controls). Exosomal miR-18a-5p expression levels were quantified by qRT-PCR (n = 10, *P < 0.05; Student’s t-test).

Having shown that inhibiting TNFα production reduced the severity of UPEC-induced cystitis, we next investigated which factors within MB49-U-Exo induced TNFα production by macrophages. Since miRNA (an important cargo in exosomes) plays a crucial role in regulation of gene expression, we next identified 326 miRNAs that were enriched in MB49-U-Exo by miRNA sequencing the contents of MB49-U-Exo and MB49-Exo (Fig 7A and S1 Table). KEGG pathway analysis revealed that the genes targeted by the most significantly enriched miRNAs were related to the MAPK signaling pathway (Fig 7B). Vivanco et al. previously demonstrated that JNK is a functional target of the phosphatase and tensin homolog (PTEN) [23]. We therefore used the TargetScan database to ascertain whether any of the miRNAs most significantly enriched in MB49-U-Exo (namely miR-130a-3p, miR-21a-5p, and miR-18a-5p) targeted PTEN; the potential PTEN binding sites of these candidate miRNAs are shown in Fig 7C. Western blot analysis confirmed that only the miR-18a-5p mimic reduced PTEN expression and activated JNK signaling in BMDMs (Figs 7D and S7). In addition, treating BMDMs with the miR-18a-5p mimic significantly upregulated their expression of *Tnfα* mRNA (Fig 7E). Although miR-130a-3p and miR-21a-5p slightly increased TNFα expression in BMDMs, neither miRNA had any significant influence on PTEN expression or JNK activation in these cells. Moreover, the level of miR-18a-5p in exosomes isolated from the urine of patients with UTIs was significantly higher than that in the exosomes isolated from the urine of healthy controls (Fig 7F). These data suggest that the miR-18a-5p encapsulated in MB49-U-Exo may play a major role in inducing TNFα production in macrophages and serve as a potential biomarker in the diagnosis of UTIs.

## Discussion

**Fig 8:**
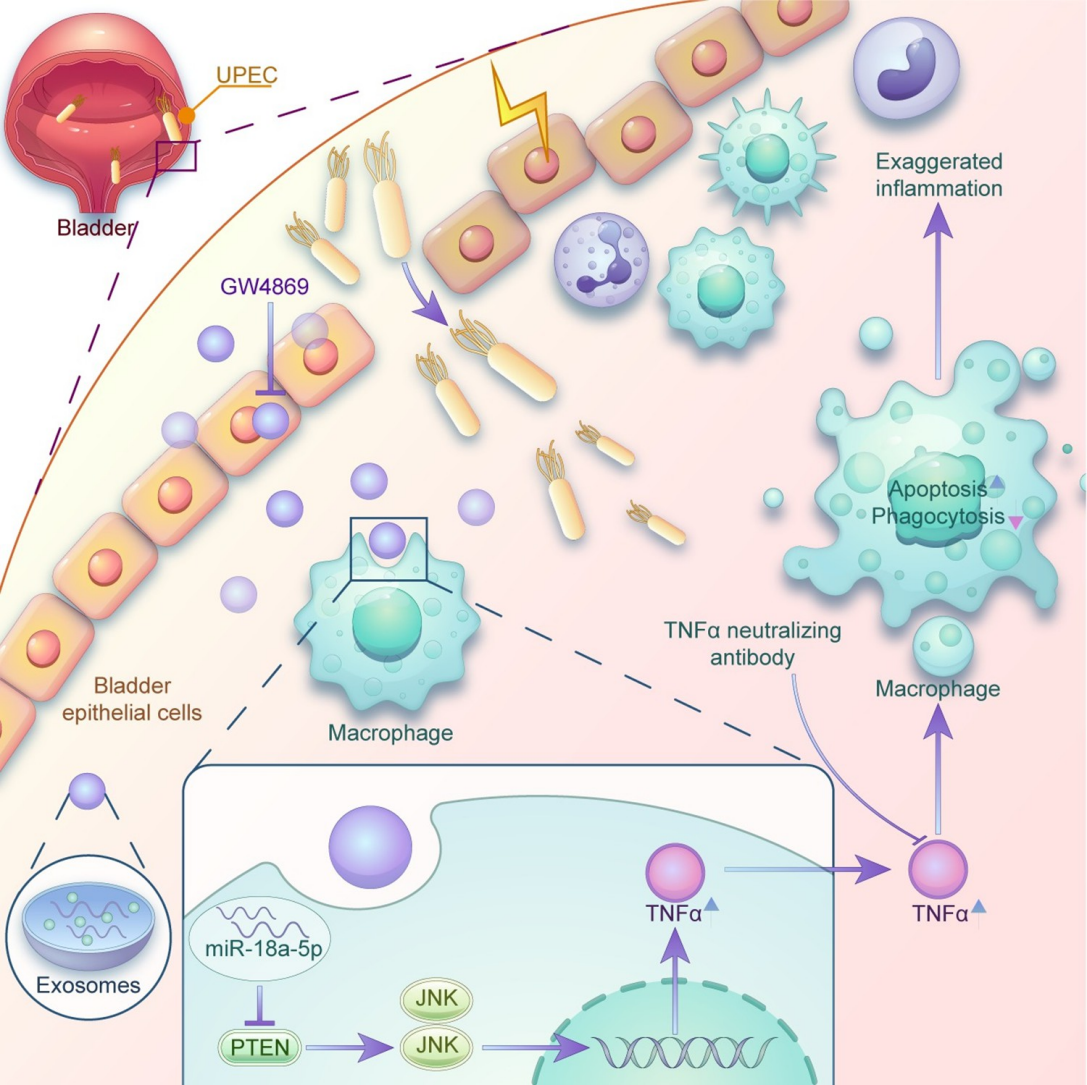
Schematic representation of exosomes derived from bladder epithelial cells after UPEC infection and their impairment of macrophage function. Exosomes released by bladder epithelial cells after UPEC infection are assimilated by macrophages, which activate their JNK signaling pathway and secret TNFα by delivering miR-18a-5p to inhibit PTEN expression, leading to macrophage apoptosis and the attenuation of phagocytosis activity. Targeting exosome release with GW4869 or a TNFα-neutralizing antibody ameliorated UPEC-elicited cystitis by decreasing macrophage and neutrophil infiltration and facilitating UPEC elimination.

The innate immune response plays an essential role in host defense against invading pathogens; however, it can also inadvertently cause tissue damage [24]. Although the innate immune response against UPEC infection has been intensively studied [7,25,26], little is known about the crosstalk between epithelial cells and immune cells that occurs during this response. In the current study, we demonstrated that miR-18a-5p encapsulated in exosomes derived from UPEC-infected bladder epithelial cells induced macrophages to produce excessive amounts of TNFα by activating the JNK pathway. This in turn promoted the apoptosis of macrophages and attenuated their phagocytic activity by downregulating the expression of genes associated with phagocytosis. We showed that inhibiting exosome release using GW4869 or a TNFα-neutralizing antibody significantly ameliorated the pathology of UPEC-inducted cystitis in mice by promoting UPEC elimination by macrophages in the bladder but preventing excessive additional macrophage and neutrophil infiltration (Fig 8). Collectively, our data show that exosome-mediated crosstalk between epithelial cells and macrophages modulates the host inflammatory response, expanding our understanding of the mechanisms underlying the regulation of the host innate immune response to UPEC infection and the pathogenesis of UPEC-associated UTIs.

Emerging evidence suggests that during UPEC infection the hyperactivation of the innate immune response leads to excessive inflammation and tissue destruction; therefore, a variety of anti-inflammatory regimens have been utilized as immunomodulation therapies for UTIs [26]. In the present study, we observed that UPEC infection induced epithelial cells to secrete a large number of exosomes, which in turn stimulated macrophages to produce excessive levels of proinflammatory cytokines, such as TNFα, inducing further bladder tissue destruction. In accordance, the exosome secretion inhibitor GW4869 significantly ameliorated the bladder pathology associated with innate immune response hyperactivation following UPEC infection, suggesting that it could potentially be used in the treatment of UTIs caused by UPEC. Similarly, COX2 inhibition was reported to attenuate bladder inflammatory responses and the extent of associated tissue destruction in a murine model of UPEC-induced cystitis [27]. Moreover, in a mouse model of UPEC-induced orchitis, corticosterone treatment lowered TNFα expression and protected the testes from inflammation-associated tissue damage by maintaining the M2 phenotype of testicular macrophages and reducing the abundance of infiltrating monocyte-derived M1 macrophages [28]. Therefore, the fine-tuning of innate immune responses to UPEC infection through, for example, the inhibition of UPEC-mediated epithelial exosome secretion may be a viable non-antibiotic strategy for treating UTIs.

Exosomes within the 40–160-nm diameter range transmit information between cells by exchanging different types of biomolecules [15]. A study has demonstrated that extracellular vesicles (EVs) derived from UPEC modulated the immune response, which may be attributed to EV-associated LPS. Meanwhile, RNA encapsulated in UPEC-derived EVs suppressed the expression of LPS-induced proinflammatory cytokines, including IL-8 and IL-1α[29]. Besides originating directly from UPEC, exosomes may also be derived from epithelial cells during UPEC infection. However, our knowledge of how UPEC infection regulates the secretion of exosomes from bladder epithelial cell and the impact of these exosomes on immune cells is lacking. It has been demonstrated that UPEC infection induced urothelial cell pyroptosis and the secretion of exosomes containing IL-1β and IL-18; this in turn led to the recruitment and activating mast cells, which ultimately damaged the urothelial barrier and impaired bladder function [30]. In addition, exosomes isolated using a mouse model of UPEC-induced orchitis promoted macrophage M1 activation and consequently increased the levels of proinflammatory cytokines (e.g., TNFα, IL-1β, and IL-6) in the testes [31]. Similarly, in the present study, we found that UPEC infection increased the release of exosomes from bladder epithelial cells. These exosomes then stimulated macrophages to secrete large amounts of TNFα, which promoted their apoptosis and impaired their phagocytic ability. The proinflammatory cytokine TNFα is produced by a variety of immune cell types, including macrophages, and is often implicated in the pathogenesis of inflammatory and autoimmune diseases [32–34]. Moreover, a prior bladder infection can influence TNFα signaling dynamics, while sustained TNFα signaling activation is associated with tissue destruction and disease severity [35]. Reminiscent of the finding that LPS induces macrophage apoptosis through the autocrine production of TNFα [36], we observed that MB49-U-Exo stimulated macrophages to secrete TNFα, which attenuated their phagocytic ability and increased their rate of apoptosis.

Because the mechanism of TNFα expression is cell-type- and stimulus-specific, it is regulated by a number of signaling pathways, including NF-κB and MAPK [37,38]. In the current study, we found that MB49-U-Exo induced TNFα production in macrophages by activating the ERK and JNK branches of the MAPK signaling pathway. JNK is a functional target of PTEN [23]. The profiling of the miRNA contents of MB49-U-Exo identified several candidates which targeted PTEN. We then showed that miR-18a-5p specifically promoted TNFα expression in macrophages by targeting PTEN and activating JNK signaling. Although miR-18a-5p has been shown to be involved in various physiological and pathological processes (e.g., cell proliferation, apoptosis, and tumorigenesis), it has mainly been studied in the context of cancer biology [39,40]. In the present study, we demonstrated that miR-18a-5p was also implicated in the pathogenesis of UPEC-induced UTIs. Similarly, miR-18a has been shown to increase insulin sensitivity by downregulating PTEN [41]. It has been suggested that Akt and CD9 in urinary exosomes could be useful markers for the diagnosis of UTIs [42]. In present study, we demonstrated that exosomal miR-18a-5p levels in the urine of patients with UTIs were significantly higher than those in the urine of healthy controls, suggesting that it could be used as a biomarker in UTI diagnosis. Collectively, these findings have helped clarify the regulatory role of exosomal miRNAs in the communication between epithelial cells and macrophages during UPEC infection of the bladder.

In summary, the findings of the present study suggest that exosomes, released from epithelial cells following UPEC infection in the bladder, modulate macrophage function to attenuate their phagocytic capacity and exacerbate inflammation, thus, reducing bacterial clearance and causing extensive tissue damage. Our results further suggest that inhibiting exosome release could be an effective, non-antibiotic strategy for treating UTIs.

One limitation of this study is that we focused on the exosomal miRNAs, and specifically those that targeted PTEN. It is therefore possible that other exosomal cargos besides PTEN-targeting miRNAs could influence macrophage function in UTI pathogenesis. Additionally, our study focused specifically on macrophages, and used female mice exclusively to model a UPEC-induced UTI and the effects of inhibiting exosome secretion on UTI pathogenesis. Further studies are needed to uncover the impact of exosomes released from UPEC-infected epithelial cells on other immune cell types (including different subsets of macrophages), and whether targeting exosome release can mitigate UTIs in both male and female subjects.

## Materials and methods

### Ethics statement

The animal experiments and the analysis of human urine samples were approved by the Ethical Review Committee of the First Affiliated Hospital of Zhengzhou University (Approval No. 2019-KY-316). All human subjects provided written informed consent before participating in this study.

### Cell culture and bacterial strains

The mouse bladder epithelial cell line MB49 and the human bladder epithelial cell line 5637 were obtained from the Chinese Academy of Sciences. MB49 cells were cultured in DMEM medium (GE Healthcare Life Sciences, Pittsburgh, PA, USA), supplemented with 10% FBS (PAN Biotech, Aidenbach, Germany), 100  IU/mL penicillin, and 100  μg/mL streptomycin (Gibco, Waltham, MA, USA), in a humidified incubator at 37°C with 5% CO_2_. The 5637 cells were cultured in RPMI-1640 medium(Cytiva, Marlborough, MA, USA)supplemented with 10% FBS, 100  IU/mL penicillin, and 100  μg/mL streptomycin. The UPEC strains 536 (National Center for Biotechnology Information: NC_008253, CP000247) and UTI89 (NC_007946, CP000243) were used in this study. UPEC 536 were transfected with the pTD103luxl_sfGFP plasmid (Addgene, Watertown, MA, USA) to introduce GFP fluorescence; and the stable GFP-expression clone was then selected using kanamycin (50 μg/mL). The bacterial strains were inoculated into LB broth and grown to early growth phase (OD600 = 0.5–0.8) at 37°C in a shaking incubator.

### Animals

C57BL/6J female mice (8–10 weeks of age) were purchased from GemPharmatech (Nanjing, China). Mice were subjected to a 12-h dark/light cycle in a room with controlled temperature (20–25°C) and humidity (40%–60%). All animals were fed laboratory standard autoclaved food in a barrier facility.

### Human urine samples

Human urine samples were obtained from 10 patients diagnosed with acute cystitis and 10 healthy donors in the First Affiliated Hospital of ZhengZhou University. Information relating to all subjects is presented in S2 Table.

### Bacterial adhesion and invasion assays

MB49 or 5637 cells were seeded in 6-well plates and infected for 1 h with UPEC at a multiplicity of infection (MOI) of 10 when the cell confluence reached ~90%. The cells were then washed five times with phosphate-buffered saline (PBS) and digested using trypsin. Next, the cells were resuspended in 100 μL PBS, serially diluted, and plated on LB agar plates. In the invasion assays, the infected cells were treated with 100 μg/mL gentamicin (Sangon Biotech, Shanghai, China) for 1 h to kill any extracellular bacteria. After three washes with PBS, the cell suspension was lysed with 0.1% Triton X-100 for 10 min. Finally, the cell lysate was plated onto LB agar plates and the number of intracellular bacteria was quantified.

### Exosome isolation

Exosomes derived from MB49 cells (MB49-Exo), 5637 cells (5637-Exo), MB49 cells infected with the UPEC strain 536 (MB49-U-Exo), 5637 cells infected with the UPEC strain 536 (5637-U-Exo), or 5637 cells infected with the UPEC strain UTI89 (5637-UTI89-Exo) were isolated from the cell culture supernatant by multi-step centrifugation. Briefly, to collect MB49-Exo and 5637-Exo, 80% confluent MB49 cells or 5637 cells were washed twice with PBS, immersed in FBS-free DMEM or RPMI-1640 respectively, and cultured for 24 h. Similarly, MB49 or 5637 cells were washed with PBS five times and then treated with UPEC strains 536 or UTI89 at an MOI of 10 for 1 h. The extracellular UPEC were washed away with PBS, and the cells were incubated with FBS-free DMEM supplemented with gentamicin (100 μg/mL) for another 24 h. The collected culture medium was filtered through a 0.22-μm syringe filter and then centrifuged at 200 × g and then at 20,000 × g (each time for 20 min at 4°C) to remove cells and cell debris, respectively. Then, the supernatant was subjected to ultracentrifugation at 100,000 × g for 70 min at 4°C on a Hitachi ultracentrifuge (CS150FNX, Tokyo, Japan). After washing with PBS, the collected exosomes were resuspended in PBS and stored at −80°C for use in subsequent experiments. The exosomes were tested for their expression of the exosomal markers protein cluster of differentiation 63 (CD63, AB_627877; Santa Cruz Biotechnology) and heat shock protein 90 (HSP90, AB_2233307; Cell Signaling Technology). The particle count and size distribution of the exosomes were also measured using the NanoSight NS300 instrument (Malvern Instruments Ltd., Malvern, UK) equipped with NTA 3.0 analytical software (Malvern Instruments Ltd.). Exosome isolation from human or mouse urine was performed using the same multi-step centrifugation protocol as described above.

### Transmission electron microscopy

A transmission electron microscope (HT7800, Hitachi, Tokyo, Japan) was used to verify exosomal structure and presence. The exosomes were dropped onto the copper net and negatively dyed with 2% phosphotungstic acid for 2 min. After being left to dry for 15 min, the MB49-Exo and MB49-U-Exo were observed under an 80-kV transmission electron microscope (HITACHI Regulus 8100, Japan).

### Lactate dehydrogenase activity assay

MB49 and 5637 cells were seeded in 96-well plates and infected with the UPEC strains 536 or UTI89 for 1 h at an MOI of 10. After five washed with PBS, the cells were transferred to a culture medium containing 100 μg/mL gentamicin. After a 24-h incubation, the plates were centrifuged at 200 × g for 5 min, and the culture medium was collected and assayed using the Cytotoxicity LDH Assay Kit-WST (Dojindo Laboratories, Kumamoto, Japan) according to the manufacturer’s protocol.

### BMDMs isolation and treatment

BMDMs were isolated from the femurs and tibias of adult C57BL/6J mice, as previously described [28]. Briefly, after the mice were euthanized, the major muscles around the femur and tibia were removed. The bone marrow was then flushed out using a 1-mL syringe filled with PBS and centrifuged at 300 × g for 5 min at 4°C. The bone marrow was treated with red blood cell lysis buffer (Solarbio, Beijing, China) for 3 min to remove the erythrocytes, before being resuspended in RPMI-1640 medium and filtered through a 70-μm cell strainer. Approximately 2 × 10^6^ cells/well were seeded onto 12-well plates and rested overnight. After washing away any unattached cells, the adherent BMDMs were cultured with RPMI-1640 medium (10% FBS, 1% penicillin, and streptomycin) and 20 ng/mL granulocyte-macrophage colony-stimulating factor (GM-CSF, Biolegend, San Diego, CA, USA). The BMDMs were treated with MB49-Exo or MB49-U-Exo at a concentration of 10 μg/mL for the indicated time periods. In some experiments, the BMDMs were also simultaneously treated with an ERK inhibitor (PD98059, 20 μM, MedChemExpress, Monmouth Junction, NJ, USA) or a JNK inhibitor (SP600125, 40 nM, MedChemExpress) in combination with MB49-U-Exo for 3 h.

### Quantitative real-time (qRT)-PCR

Total mRNA was extracted from cells using RNAiso Plus (9109, Takara, Tokyo, Japan), and reverse-transcribed into cDNA using the Prime Script RT Master Mix (RR036A; Takara). For the quantitative miRNA analysis, total miRNA was extracted from exosomes using the miRNeasy Mini Kit (217004, Qiagen, Hilden, Germany), and reverse transcribed using the miRNA First Strand cDNA Synthesis Kit (B532453, Sangon Biotech), which uses stem-loop method. qRT-PCR was performed using the SYBR Green PCR Master Mix (Application Takara, Otsu, Japan) on the ABI Prism 7300HT sequence detection system (Applied Biosystems, Foster, CA, USA). The relative expression of each gene was calculated using the 2^−ΔΔCt^ method; *Actin* and U6 were used as internal controls for mRNA and miRNA expression, respectively. The sequences of primers used in this study are listed in S3 Table.

### Mouse cytokine detection assay

Supernatant was isolated from the culture medium of BMDMs stimulated with MB49-Exo or MB49-U-Exo for 12 h. Cytokine production by the exosome-treated BMDMs was detected using the mouse multiple cytokines ELISA kit (LX-MultiDTM-23, Labex, Shanghai, China), according to the manufacturer’s instructions.

### Flow cytometry

To detect immune cell populations in the bladder, the bladders of mice with UPEC-induced cystitis were harvested and digested with 0.1 mg/ml type D collagenase (StemCell Technologies, Vancouver, BC, Canada) for 30 min at 37°C. The digested cell suspensions were filtered through a 70-μm cell strainer. Single cells were washed twice with PBS and stained with antibodies against: CD45 (APC-R700, Biolegend), F4/80 (BV-605, Biolegend), CD11b (APC-Cy7, Biolegend), CD64 (APC, Biolegend), and Ly6G (PE, Biolegend). The macrophage and neutrophil populations were detected using a flow cytometer (BD Canto, Franklin Lakes, NJ, USA). Data were analyzed using FlowJo X software (Tree Star, Ashland, OR, USA).

Following treatment with MB49-Exo/MB49-U-Exo (10 μg/mL) or a TNFα-neutralizing antibody (Cell Signaling Technology, MA, USA) at a concentration of 100 ng/mL for 12 h, the BMDMs were washed twice with PBS and detached using trypsin-EDTA. Apoptosis was evaluated using the Annexin V (FITC)/PI apoptosis detection kit (BioLegend) according to the manufacturer’s instruction. The population of Annexin V^+^ or PI^+^ cells was detected by flow cytometry. All Annexin V^+^ cells, irrespective of their PI expression were considered to be apoptotic.

### RNA interference

The miR-130-3p, miR-21a-5p, and miR-18a-5p mimics and their respective negative controls were obtained from Sangon Biotech (Shanghai, China). The sequences of the miRNAs are listed in S4 Table. BMDMs were transfected with the miRNAs using INTERFERin (Illkirch, France) according to the manufacturer’s instructions.

### Phagocytosis experiment

BMDMs were treated with MB49-Exo/MB49-U-Exo at a concentration of 10 μg/mL for 24 h and then incubated with EGFP-UPEC or FITC-labelled *E*. *coli* BioParticles (Invitrogen, Carlsbad, CA, USA) at 37°C for 1 h. After washing with cold PBS, the cells were immediately collected, resuspended in staining buffer (PBS containing 1% bovine serum albumin), and stained with anti-CD45 (APC-R700, Biolegend), anti-F4/80 (BV-605, Biolegend), and anti-CD11b (APC, Biolegend) antibodies. The cell suspension was then acquired on a flow cytometer (BD Canto) to measure the green fluorescence of CD45^+^F4/80^+^CD11b^+^cells. Data were analyzed using FlowJo X software (Tree Star).

### Coculture assays

To explore the effect of exosomes derived from bladder epithelial cells on the phagocytic ability of macrophages, MB49 cells and BMDMs were cocultured in a Transwell culture system; the BMDMs were seeded into the bottom of a 12-well plate while the MB49 cells were seeded into the 12-well culture inserts. Prior to coculture, the MB49 cells were incubated with UPEC strain 536 (MOI: 10) or PBS for 1 h. The extracellular UPEC were then washed away with PBS, and the cells were incubated with FBS-free DMEM supplemented with gentamicin (100 μg/mL) for another 2 h. The pretreated MB49 cells were then cocultured with BMDMs by transferring the culture inserts into a 12-well plate containing the BMDMs. In some experiments, the cells were treated with GW4869 (10 μg/mL, MedChemExpress, NJ, USA) or a TNFα-neutralizing antibody (100 ng/mL) for 12 h. Similarly, THP1 cells were placed in the bottom well of a Transwell system and cocultured with 5637 cells (located in the upper well), which were pretreated with UPEC 536 or PBS. In some experiments, the cells were treated with GW4869 or infliximab (100 μg/mL, Meilunbio, Dalian, China) for 12 h. Finally, the upper culture inserts were removed and the BMDMs or THP1 cells were incubated with FITC-labelled *E*. *coli* BioParticles (50 μg/mL) for 1 h, before their phagocytic activities was assessed by flow cytometry.

### Exosome tracking in vitro and in vivo

To identify the uptake of MB49-Exo and MB49-U-Exo by BMDMs, as well as that of 5637-Exo, 5637-U-Exo, and 5637-UTI89-Exo by THP1 cells, the isolated exosomes were labeled with the Cy5.5 fluorescent dye (Aladdin, Shanghai, China) at a working concentration of 0.1 mg/mL for 30 min at 37°C. The labeled exosomes were washed with PBS and centrifuged at 100,000 × g, 4°C for 70 min to remove the residual dye. BMDMs or THP1 cells were incubated with Cy5.5-labeled exosomes (10 μg/mL) for 1 h at 37°C in the dark. Next, the internalization of exosomes was observed with a laser-scanning confocal microscope or analyzed by flow cytometry.

For tracking the uptake of exosomes in vivo, Cy5.5-labeled exosomes (1 μg in 100 μL PBS) or unlabeled exosomes (PBS control) were injected into the mouse bladder via the urethra. After 12 h, the entire bladder was harvested, chopped using scissors, and digested with collagenase D (0.1 mg/mL) and DNase I (100 U/mL) (both from Sigma-Aldrich) for 30 min at 37°C to obtain a single-cell suspension. The percentage of CD45^+^F4/80^+^ bladder macrophages within the Cy5.5^+^ cell population was analyzed by flow cytometry (BD Canto).

### Western blotting

Cells or exosomes were harvested at the indicated times and lysed using standard cell lysis buffer (Solarbio, Beijing, China) on ice for 30 min. Protein concentrations were determined using the Pierce BCA Protein Assay Kit (Beijing Leagene Biotech Co. Ltd.). Proteins (20 μg) were separated using SDS-PAGE 10% polyacrylamide gels and transferred onto PVDF membranes (Merck Millipore, USA). The blots were blocked with 5% skimmed milk for 1 h at room temperature and incubated with primary antibodies (listed in S5 Table) overnight at 4°C. Thereafter, the membranes were incubated with peroxidase-conjugated secondary antibodies at room temperature for 1 h, and the membrane blots were developed using an enhanced chemiluminescence kit (Beyotime Institute of Biotechnology); this was followed by chemiluminescence detection using an imaging system (Bio-Rad Laboratories, Inc., Hercules, CA, USA). After stripping the phosphorylated proteins, the membranes were blocked and reprobed with primary antibodies specific to the corresponding total proteins. The relative levels of p-ERK, p-P38, p-JNK, and p-P65 were determined by normalization to total protein expression, and the relative level of PTEN was normalized to β-actin; the intensity of the individual protein bands was measured using ImageJ software (NIH, Rasband, WS, USA). Details of all the antibodies used in this study are provided in S5 Table.

### ELISA

Murine TNFα protein production was detected in cell-free culture supernatants by ELISA according to the manufacturer’s instructions (Biolegend). The absorbance was measured at 450 nm, and the cytokine concentration (expressed as pg/mL) was determined using a standard curve.

The LPS content of MB49-Exo and MB49-U-Exo was determined using the LPS ELISA kit (Yuanju Biotechnology Center, Shanghai, China) according to the manufacturer’s instructions. The absorbance was measured at 450 nm and the LPS concentration (expressed as EU/L) was determined using a standard curve.

### Immunofluorescence staining

To track EGFP-labelled UPEC, as well as other immunofluorescent markers, mouse bladders were frozen in optimum cutting temperature medium (OCT, Sakura Finetek, Inc., Torrance, CA, USA), cut into 6-μm slices, and fixed with precooled acetone for 20 min. Next, the slices were sealed with 5% normal donkey serum for 60 min at room temperature and incubated with a fluorescently-labelled primary antibody against F4/80 (1:200, Cell Signaling Technology). An Alexa-Fluor-555-conjugated donkey anti-rabbit IgG was used as the secondary antibody. After washing, the nuclei were stained with DAPI for 10 min at room temperature. Images were captured on a Vectra machine (PerkinElmer, Waltham, MA, USA).

### RNA-sequencing

To identify the gene expression profile of BMDMs stimulated with MB49-Exo or MB49-U-Exo, RNA sequencing was performed by OE Biotech Co., Ltd. (Shanghai, China). Briefly, RNA was isolated from BMDMs, which were incubated with 10 μg/mL MB49-Exo or MB49-U-Exo for 3 h, and collected in RNAiso Plus reagent (9109, Takara, Tokyo, Japan). These RNA samples were then converted into libraries of cDNA fragments using the TruSeq Stranded mRNA LTSample Prep Kit (Illumina, San Diego, CA, USA) according to the manufacturer’s instructions. Next, these libraries were sequenced on an Illumina sequencing platform (HiSeq 2500 or Illumina HiSeq X Ten), and 125-bp/150-bp paired-end reads were generated. The data were analyzed using EdgeR software, and the statistical significance threshold for differential gene expression was defined as P < 0.05. The RNA sequencing data were uploaded to the Sequence Read Archive (SRA) database: PRJNA884531.

### miRNA microarray

Microarray analysis using the Agilent Mouse miRNA Microarray Kit, Release 21.0, 8 × 60 K (Design ID: 070155; Agilent Technologies, California, USA), was conducted by OE Biotechnology Co., Ltd. Sample labeling, microarray hybridization, and washing were performed according to the manufacturer’s instructions (Agilent Technologies). Feature Extraction Software (version 10.7.1.1, Agilent Technologies) was used to further analyze the collected array images. The target genes of the differentially expressed miRNAs in MB49-Exo and MB49-U-Exo were predicted using the miRDB (https://mirdb.org/) and miRWalk (http://mirwalk.umm.uni-heidelberg.de/) databases. Potential sites of miRNA to mRNA binding were analyzed using the TargetScan online matching tool (https://www.targetscan.org/vert_80/).

### Animal experiments

To evaluate the potential effect of exosomes on the pathogenesis of UPEC-associated UTIs, we established a murine model of UPEC-induced cystitis as previously described [43,44]. Briefly, the mice first received an injection of MB49-Exo or MB49-U-Exo (1 μg in 100 μL PBS) via the urethra; the control mice received the same volume of PBS. After 24 h, the bladders of mice were injected with 50 μL PBS containing 1 ×10^8^ CFU UPEC or 50 μL PBS (control), again via the urethra. Mice were sacrificed at 2 days post-infection, and the bladders were harvested for flow cytometric and pathological analysis.

To evaluate the effect of blocking exosome secretion or neutralizing TNFα on the severity of UPEC-induced cystitis, we treated the model mice with GW4869 or a TNFα-neutralizing antibody. In these experiments, the C57BL/6J mice received a transurethral injection of PBS or 1 × 10^8^ CFU EGFP-UPEC, followed by a daily intraperitoneal injection of GW4869 (2.5 μg/g) or the TNFα-neutralizing antibody (0.2 μg/g). On day 3, the mice were sacrificed and their bladders were harvested for flow cytometric analysis, pathological analysis, or bacteria enumeration. For the latter, the bladders were homogenized in sterile PBS, serially diluted, and then plated onto LB agar. Each bladder section was stained with H&E and scored (using a semi-quantitative scoring system) by two independent pathologists, as previously described [44–46]. The following histopathological grading scale was used: 0, normal; 1, focal and multifocal subepithelial inflammatory cell infiltration; 2, edema and diffuse subepithelial inflammatory cell infiltration; 3, marked subepithelial inflammatory cell and neutrophil infiltration into the bladder mucosal epithelium, with evidence of necrosis; 4, inflammatory cell infiltration, extending into the smooth muscle layer (assessed on the basis of grade 3 criteria); 5, loss of surface epithelium, characterized by necrosis with full-thickness inflammatory cell infiltration.

### Statistical analysis

All experimental data were processed and analyzed using Prism 8 software (Graphpad Software, Inc.). Comparisons between two groups were performed using the independent sample t-test with Welch correction. One-way or two-way ANOVA was used to compare normally distributed data from multiple groups. Mann–Whitney *U* test and Kruskal–Wallis test were used for results with nonnormal distribution like bacterial CFU. Data were displayed as the mean ± standard deviation (SD). P-values < 0.05 were considered as a measure of statistical significance.

## Supporting information

S1 Fig Macrophages take up exosomes. (A) After BMDMs were treated with Cy5.5-labeled exosomes, immunofluorescence staining showed the uptake of exosomes (pink) in CD11b^+^ (green) macrophages. DAPI was used to stain nuclei (blue), and images were captured by confocal microscopy. Scale bar = 20 μm. (B-C) Adhesion (B) and invasion assays (C) involving the UPEC strains 536 and UTI89. MB49 cells and 5637 cells were separately infected with bacteria at an MOI of 10 (n = 3; ns: no significance; two-way ANOVA). Bar graphs show the numbers of adherent and intracellular bacteria, which were determined by plating serial dilutions of bacteria onto LB agar plates. (D) Cytotoxicity LDH assay of 5637 and MB49 cells infected with UPEC 536 or UTI89 for 24 h. Bar graphs showing the relative percentage change of LDH levels in infected cells compared with that of uninfected cells (n = 3; ns: no significance; two-way ANOVA). (E) Size distribution of exosomes from uninfected (5637-Exo) and UPEC-infected (5637-UPEC-Exo) 5637 cells. (F) Representative western blots showing the expression of CD63 and HSP90 in 5637 exosomes. (G) Flow cytometric analysis of Cy5.5 MFI of THP1 cells following treatment with Cy5.5-labeled exosomes or PBS (control) at the indicated concentration for 3 h. Histogram and bar graphs showing the Cy5.5 MFI of THP1 cells after exosome absorption (n = 3; ns: no significance; Student’s t-test).
(TIF)

S2 Fig Differential gene expression in MB49-U-Exo- vs. MB49-Exo-treated BMDMs. (A) Concentration of LPS in MB49-Exo and MB49-U-Exo, measured by ELISA. Bar graph showing the OD450 of the different groups (n = 5; ns: no significance; one-way ANOVA). (B) The gene expression profiles of BMDMs treated with MB49-Exo or MB49-U-Exo for 3 h were determined by RNA sequencing analysis. Volcano plot demonstrating the magnitude and significance of genes that were upregulated (red) or downregulated (green) in MB49-U-Exo- vs. MB49-Exo-treated BMDMs. (C) The KEGG pathway map of differentially expressed genes in BMDMs following indicated exosome treatment.
(TIF)

S3 Fig Quantification of western blot data for Fig 4B–4D. (A) Bar charts showing the ratios of phosphorylated to total ERK, P38, JNK, and P65 in BMDMs treated with MB49-U-Exo for the indicated time periods (n = 3; ns: no significance, *P < 0.05, **P < 0.01, ***P < 0.001; one-way ANOVA). (B) The relative p-ERK/ERK protein ratios in BMDMs stimulated with either PBS, PD98059 (20 μM), MB49-U-Exo, or a combination of MB49-U-Exo and PD98059 for 3 h (n = 3; ns: no significance, **P < 0.01, ***P < 0.001; one-way ANOVA). (C) Bar charts showing the protein ratios of p-JNK/JNK in BMDMs stimulated with either PBS, SP600125 (40 nM), MB49-U-Exo, or a combination of MB49-U-Exo and SP600125 for 3 h (n = 3; ns: no significance, **P < 0.01, ***P < 0.001; one-way ANOVA).
(TIF)

S4 Fig MB49-Exo activate the MAPK/ERK signaling pathway in BMDMs. Western blot and bar charts showing expression of phosphorylated and total ERK, P38, JNK, and P65 in BMDMs treated with PBS or MB49-Exo for indicated time periods. (n = 3; ns: no significance, *P < 0.05, **P < 0.01; one-way ANOVA).
(TIF)

S5 Fig Exosomes derived from UPEC-infected 5637 cells suppress the phagocytic activity of macrophages. THP1 cells (as exosome recipients) were placed in the bottom well of a Transwell system and cocultured with UPEC-infected or uninfected 5637 cells (as exosome donors), placed in the upper inserts. Cells were treated with GW4869 (10 μg/mL) or infliximab (100 μg/mL) for 12 h. The cocultured THP1 cells were incubated with FITC-labelled *E*. *coli* BioParticles (50 μg/mL) for 1 h, and their phagocytic activity was assessed by flow cytometry. Flow cytometry plots show the gating strategy. Bar graphs show the FITC-positive cell populations in each group (n = 3; **P < 0.01, ***P < 0.001; one-way ANOVA).
(TIF)

S6 Fig Inhibition of TNFα production reduces bacterial burden and macrophage infiltration in the bladder. (A-B) Mice with UPEC-induced cystitis were treated with either GW4869 (2.5 μg/g) or a TNFα-neutralizing antibody (0.2 μg/g) for 3 days. Immunofluorescence staining was then used to determine the distribution of F4/80^+^ macrophages (red) (A) and bacteria (green) (B) in the bladder. Scale bar = 200 μm.
(TIF)

S7 Fig Quantification of PTEN and p-JNK expression. Bar charts showing the ratios of PTEN to β-actin, and phosphorylated JNK to total protein in BMDMs transfected with the miRNA mimics for 6 h (n = 3; ns: no significance, *P < 0.05, **P < 0.01; one-way ANOVA).
(TIF)

S1 Table Differential expressed miRNAs list. (DOCX)

S2 Table Information for human subjects. (DOCX)

S3 Table Primers used for qRT-PCR. (DOCX)

S4 Table Sequences of miRNA mimics. (DOCX)

S5 Table Antibody list. (DOCX)
