# Supplementary figures and images for: Exosomes derived from bladder epithelial cells infected with uropathogenic Escherichia coli increase the severity of urinary tract infections (UTIs) by impairing macrophage function
Source: PLoS Pathog. 2024 Jan 8;20(1):e1011926. doi: 10.1371/journal.ppat.1011926 (PMC10798623; doi:10.1371/journal.ppat.1011926)

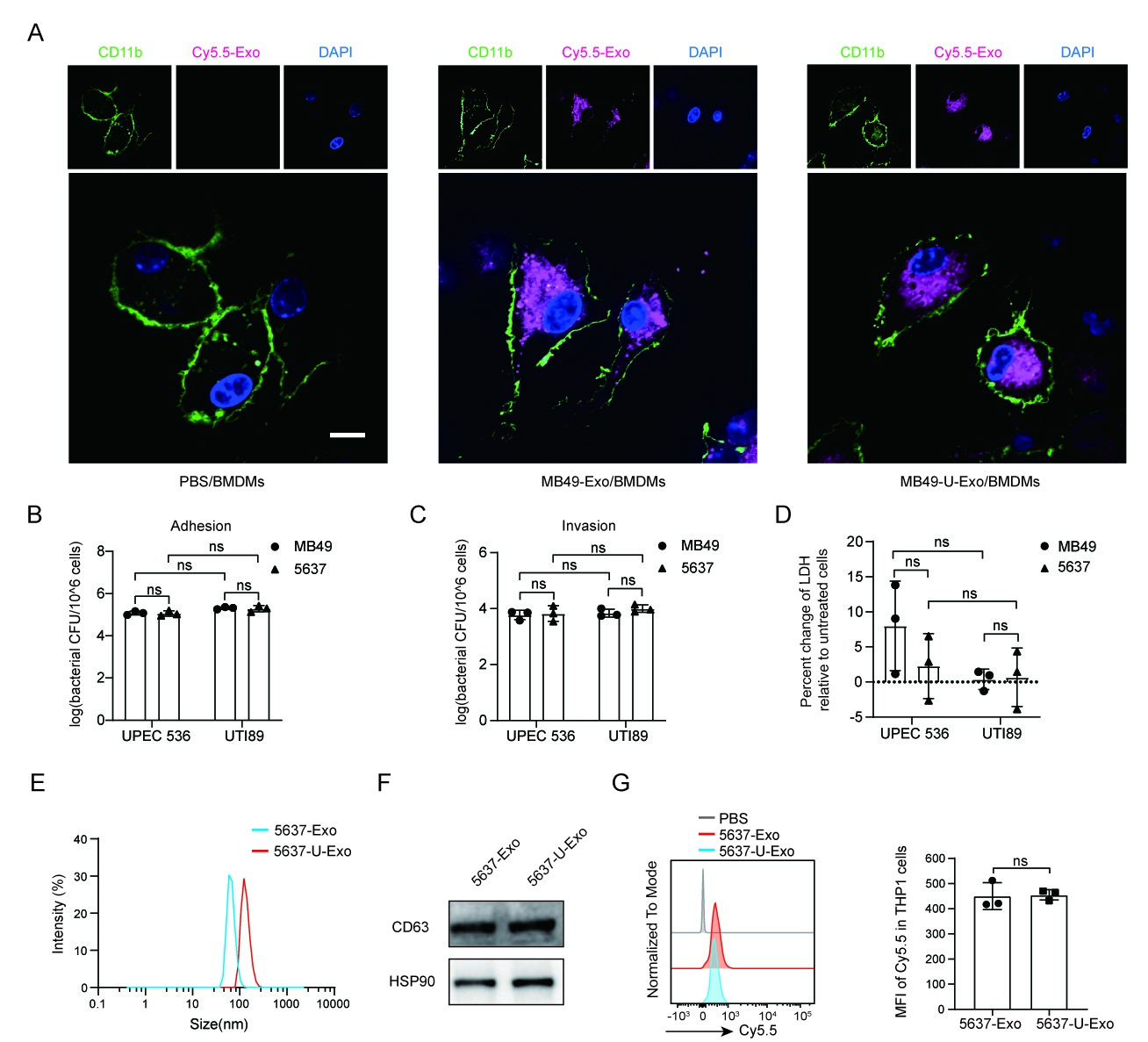

Supplement: S1 Fig — (A) After BMDMs were treated with Cy5.5-labeled exosomes, immunofluorescence staining showed the uptake of exosomes (pink) in CD11b+ (green) macrophages. DAPI was used to stain nuclei (blue), and images were captured by confocal microscopy. Scale bar = 20 μm. (B-C) Adhesion (B) and invasion assays (C) involving the UPEC strains 536 and UTI89. MB49 cells and 5637 cells were separately infected with bacteria at an MOI of 10 (n = 3; ns: no significance; two-way ANOVA). Bar graphs show the numbers of adherent and intracellular bacteria, which were determined by plating serial dilutions of bacteria onto LB agar plates. (D) Cytotoxicity LDH assay of 5637 and MB49 cells infected with UPEC 536 or UTI89 for 24 h. Bar graphs showing the relative percentage change of LDH levels in infected cells compared with that of uninfected cells (n = 3; ns: no significance; two-way ANOVA). (E) Size distribution of exosomes from uninfected (5637-Exo) and UPEC-infected (5637-UPEC-Exo) 5637 cells. (F) Representative western blots showing the expression of CD63 and HSP90 in 5637 exosomes. (G) Flow cytometric analysis of Cy5.5 MFI of THP1 cells following treatment with Cy5.5-labeled exosomes or PBS (control) at the indicated concentration for 3 h. Histogram and bar graphs showing the Cy5.5 MFI of THP1 cells after exosome absorption (n = 3; ns: no significance; Student’s t-test). (TIF) [file ppat.1011926.s001.tif]

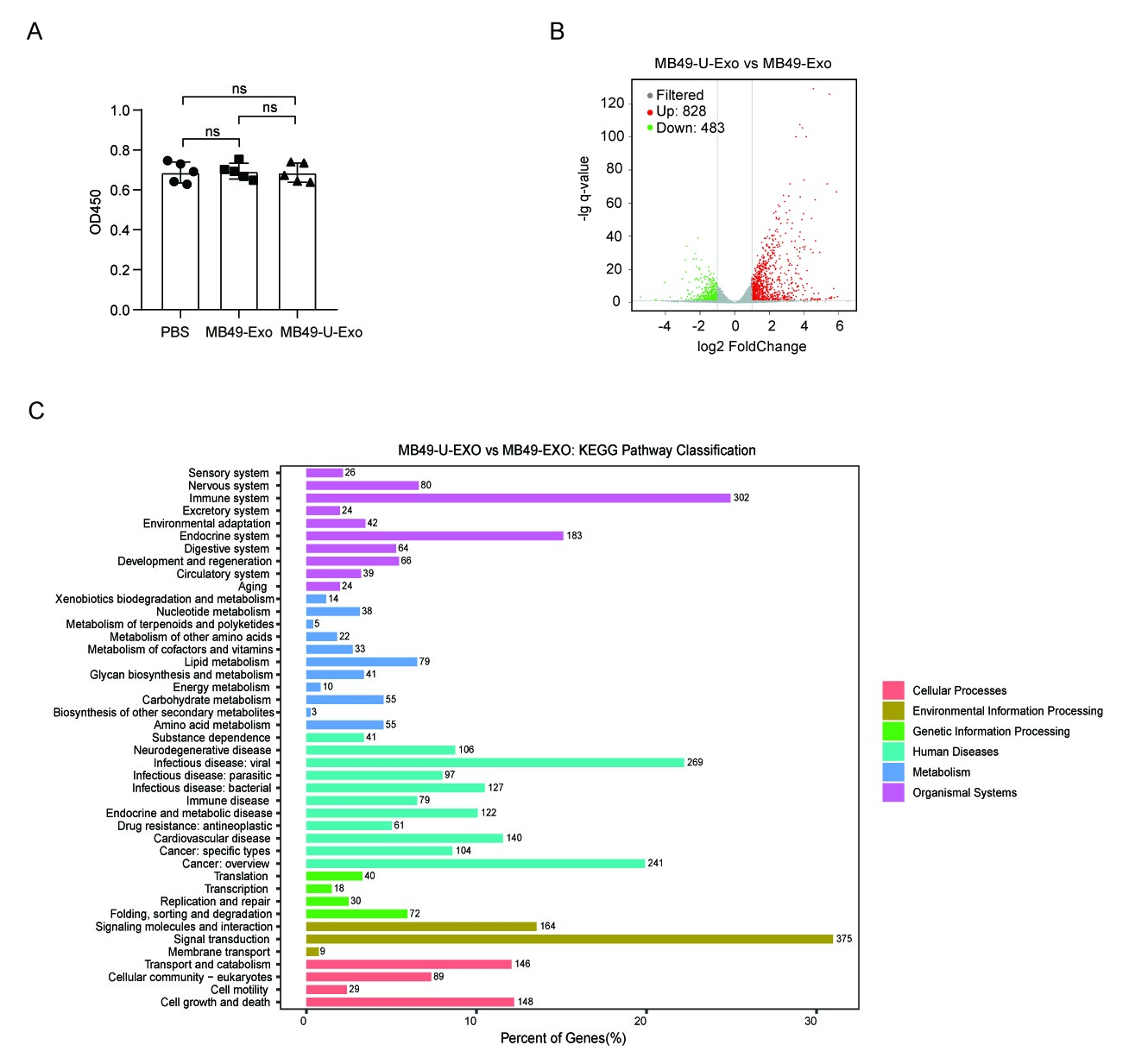

Supplement: S2 Fig — (A) Concentration of LPS in MB49-Exo and MB49-U-Exo, measured by ELISA. Bar graph showing the OD450 of the different groups (n = 5; ns: no significance; one-way ANOVA). (B) The gene expression profiles of BMDMs treated with MB49-Exo or MB49-U-Exo for 3 h were determined by RNA sequencing analysis. Volcano plot demonstrating the magnitude and significance of genes that were upregulated (red) or downregulated (green) in MB49-U-Exo- vs. MB49-Exo-treated BMDMs. (C) The KEGG pathway map of differentially expressed genes in BMDMs following indicated exosome treatment. (TIF) [file ppat.1011926.s002.tif]

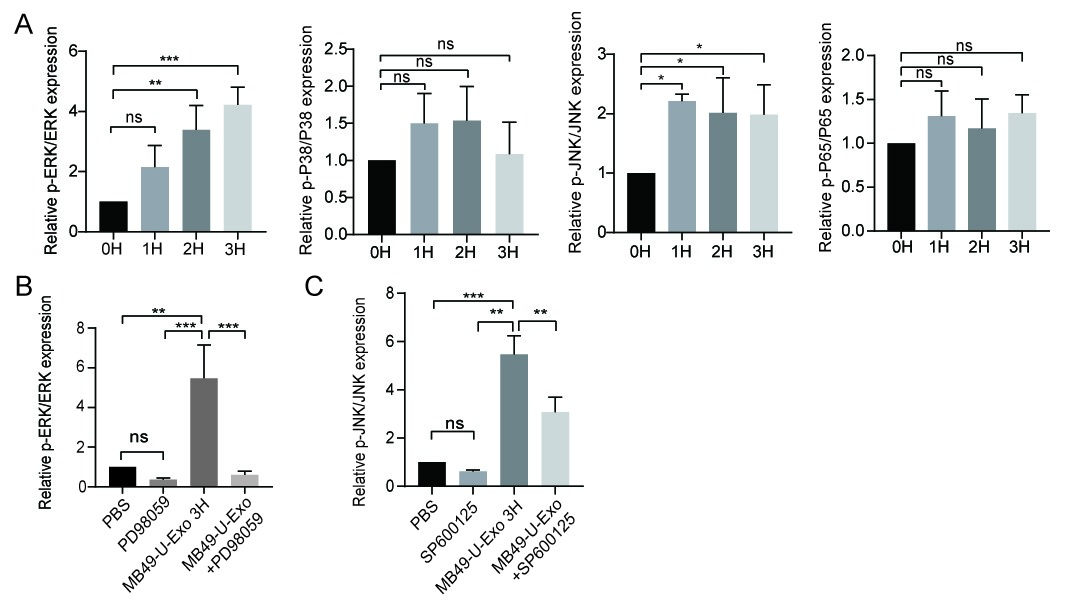

Supplement: S3 Fig — (A) Bar charts showing the ratios of phosphorylated to total ERK, P38, JNK, and P65 in BMDMs treated with MB49-U-Exo for the indicated time periods (n = 3; ns: no significance, *P < 0.05, **P < 0.01, ***P < 0.001; one-way ANOVA). (B) The relative p-ERK/ERK protein ratios in BMDMs stimulated with either PBS, PD98059 (20 μM), MB49-U-Exo, or a combination of MB49-U-Exo and PD98059 for 3 h (n = 3; ns: no significance, **P < 0.01, ***P < 0.001; one-way ANOVA). (C) Bar charts showing the protein ratios of p-JNK/JNK in BMDMs stimulated with either PBS, SP600125 (40 nM), MB49-U-Exo, or a combination of MB49-U-Exo and SP600125 for 3 h (n = 3; ns: no significance, **P < 0.01, ***P < 0.001; one-way ANOVA). (TIF) [file ppat.1011926.s003.tif]

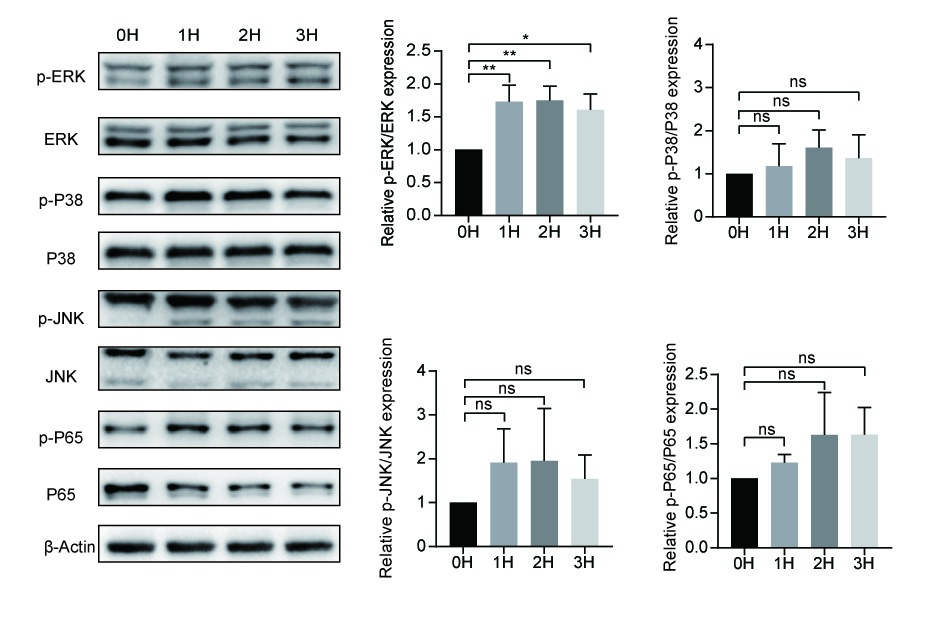

Supplement: S4 Fig — Western blot and bar charts showing expression of phosphorylated and total ERK, P38, JNK, and P65 in BMDMs treated with PBS or MB49-Exo for indicated time periods. (n = 3; ns: no significance, *P < 0.05, **P < 0.01; one-way ANOVA). (TIF) [file ppat.1011926.s004.tif]

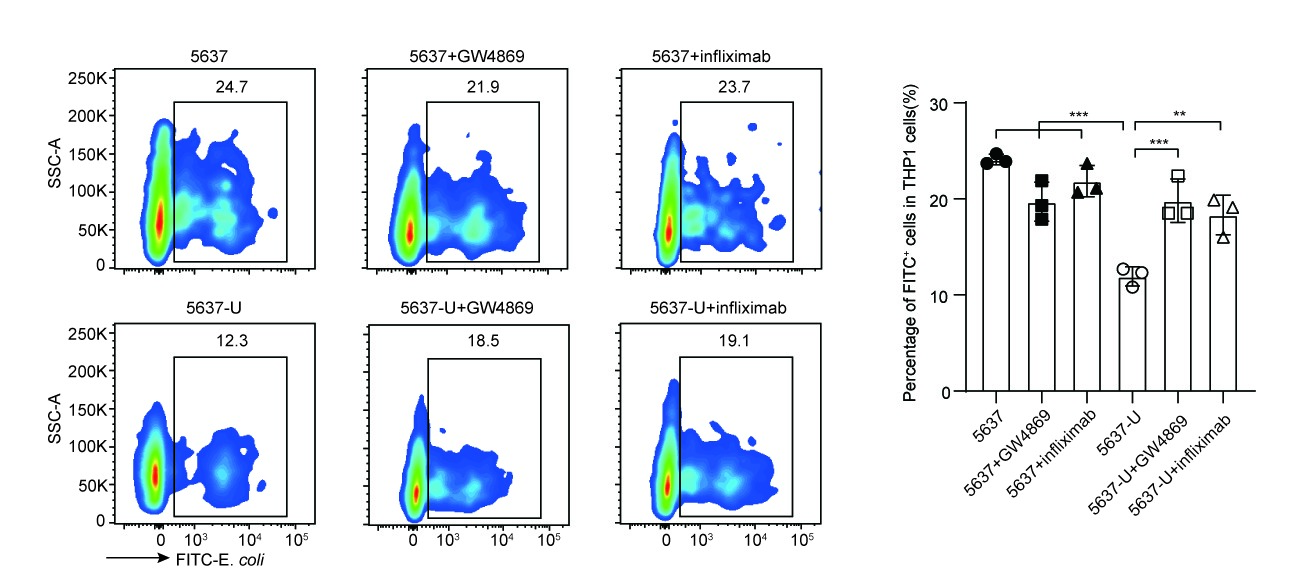

Supplement: S5 Fig — THP1 cells (as exosome recipients) were placed in the bottom well of a Transwell system and cocultured with UPEC-infected or uninfected 5637 cells (as exosome donors), placed in the upper inserts. Cells were treated with GW4869 (10 μg/mL) or infliximab (100 μg/mL) for 12 h. The cocultured THP1 cells were incubated with FITC-labelled E. coli BioParticles (50 μg/mL) for 1 h, and their phagocytic activity was assessed by flow cytometry. Flow cytometry plots show the gating strategy. Bar graphs show the FITC-positive cell populations in each group (n = 3; **P < 0.01, ***P < 0.001; one-way ANOVA). (TIF) [file ppat.1011926.s005.tif]

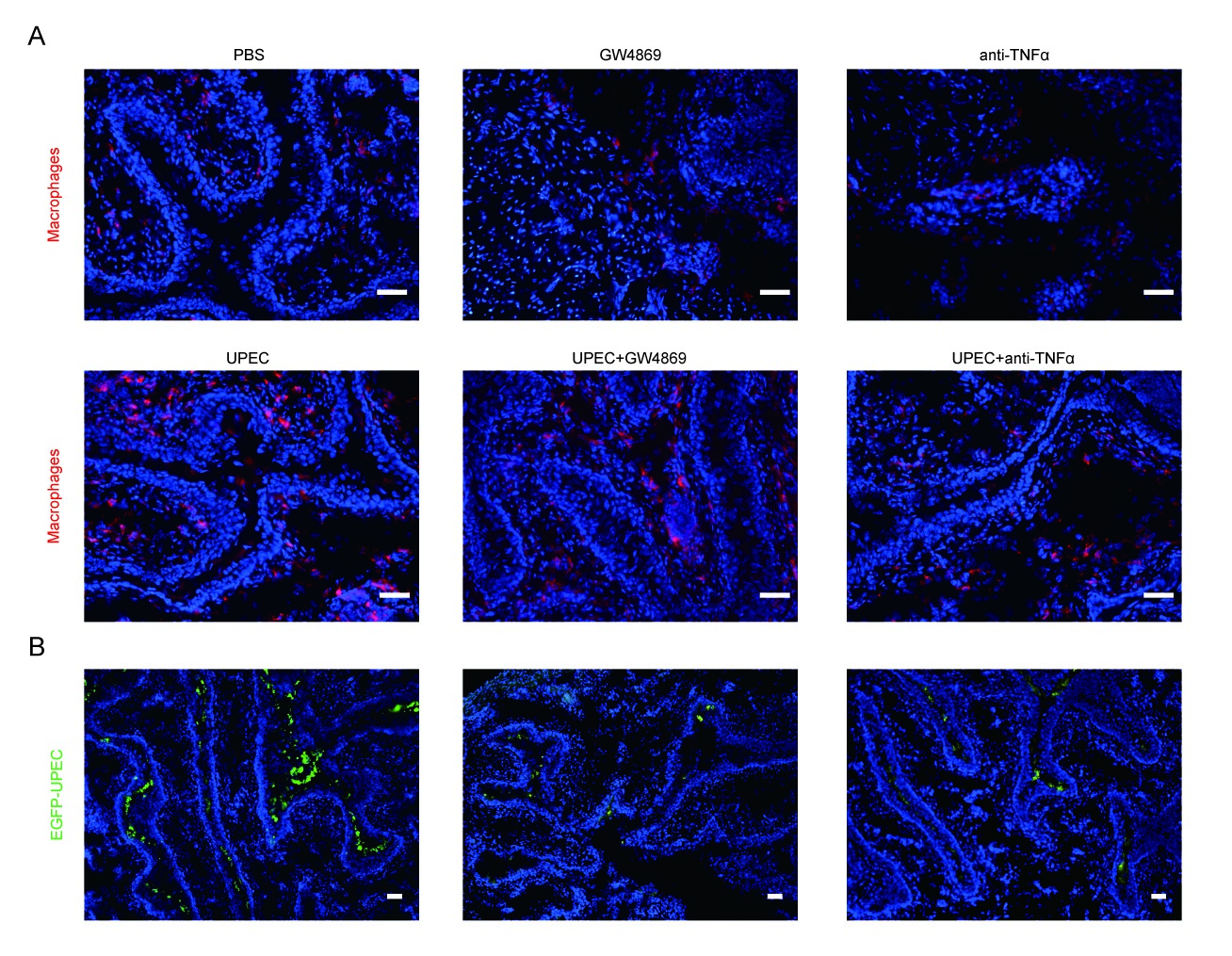

Supplement: S6 Fig — (A-B) Mice with UPEC-induced cystitis were treated with either GW4869 (2.5 μg/g) or a TNFα-neutralizing antibody (0.2 μg/g) for 3 days. Immunofluorescence staining was then used to determine the distribution of F4/80+ macrophages (red) (A) and bacteria (green) (B) in the bladder. Scale bar = 200 μm. (TIF) [file ppat.1011926.s006.tif]

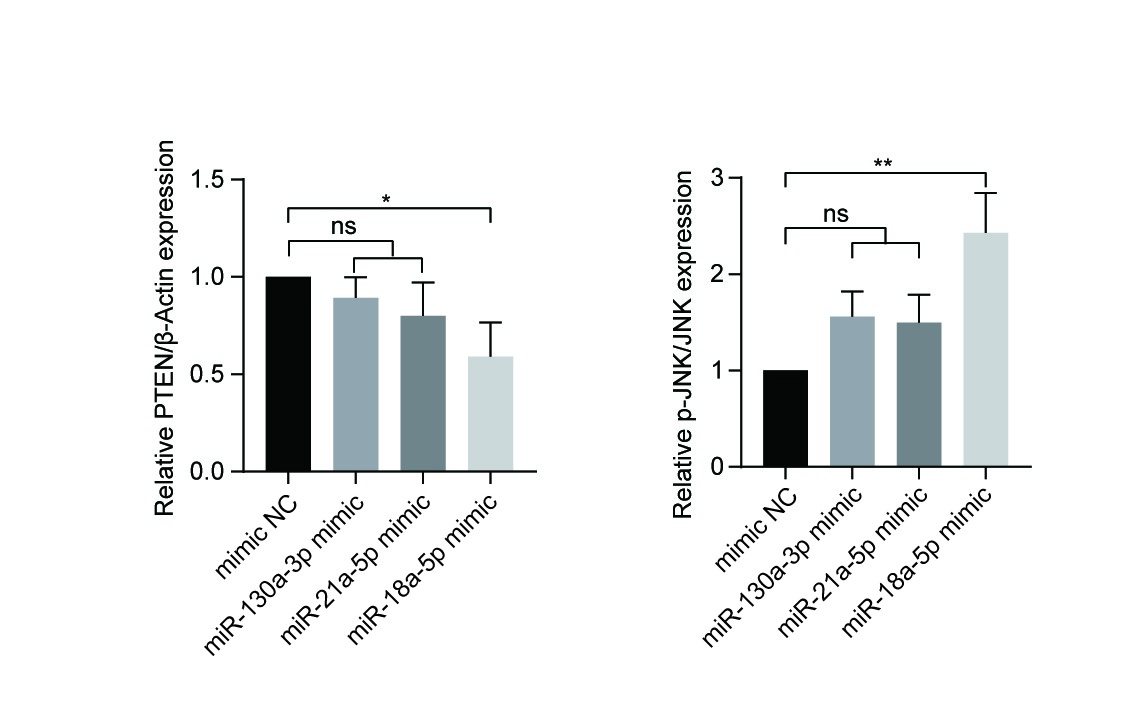

Supplement: S7 Fig — Bar charts showing the ratios of PTEN to β-actin, and phosphorylated JNK to total protein in BMDMs transfected with the miRNA mimics for 6 h (n = 3; ns: no significance, *P < 0.05, **P < 0.01; one-way ANOVA). (TIF) [file ppat.1011926.s007.tif]
