# Supplementary material for: Exosomes derived from bladder epithelial cells infected with uropathogenic Escherichia coli increase the severity of urinary tract infections (UTIs) by impairing macrophage function
Source: PLoS Pathog. 2024 Jan 8;20(1):e1011926. doi: 10.1371/journal.ppat.1011926 (PMC10798623; doi:10.1371/journal.ppat.1011926)
Supplement: S1 Table — (DOCX) [file ppat.1011926.s008.docx]

**S1 Table. Differential expressed miRNAs list**

| **miRNAs** | **Regulation(MB49-U-Exos vs. MB49-Exos)** |
| --- | --- |
| mmu-let-7g-5p | Up |
| mmu-let-7i-5p | Up |
| mmu-miR-15b-5p | Up |
| mmu-miR-23b-3p | Up |
| mmu-miR-27b-3p | Up |
| mmu-miR-29b-3p | Up |
| mmu-miR-30a-5p | Up |
| mmu-miR-99b-5p | Up |
| mmu-miR-101a-3p | Up |
| mmu-miR-125a-5p | Up |
| mmu-miR-125b-5p | Up |
| mmu-miR-126a-3p | Up |
| mmu-miR-128-3p | Up |
| mmu-miR-130a-3p | Up |
| mmu-miR-134-5p | Down |
| mmu-miR-138-5p | Down |
| mmu-miR-140-5p | Up |
| mmu-miR-140-3p | Up |
| mmu-miR-142a-3p | Up |
| mmu-miR-146a-5p | Up |
| mmu-miR-152-3p | Up |
| mmu-miR-182-5p | Up |
| mmu-miR-183-5p | Up |
| mmu-miR-184-3p | Up |
| mmu-miR-185-5p | Up |
| mmu-miR-188-5p | Down |
| mmu-miR-24-3p | Up |
| mmu-miR-193a-3p | Up |
| mmu-miR-194-5p | Up |
| mmu-miR-195a-5p | Up |
| mmu-miR-200b-3p | Up |
| mmu-miR-202-3p | Up |
| mmu-miR-30e-5p | Up |
| mmu-miR-290a-5p | Down |
| mmu-miR-291a-5p | Down |
| mmu-miR-292a-5p | Down |
| mmu-miR-296-5p | Down |
| mmu-miR-301a-3p | Up |
| mmu-let-7d-5p | Up |
| mmu-miR-106b-5p | Up |
| mmu-miR-130b-3p | Up |
| mmu-miR-19b-3p | Up |
| mmu-miR-30c-5p | Up |
| mmu-miR-30d-5p | Up |
| mmu-miR-148a-3p | Up |
| mmu-miR-192-5p | Up |
| mmu-miR-200a-3p | Up |
| mmu-let-7a-5p | Up |
| mmu-let-7b-5p | Up |
| mmu-let-7c-5p | Up |
| mmu-let-7e-5p | Up |
| mmu-let-7f-5p | Up |
| mmu-miR-15a-5p | Up |
| mmu-miR-16-5p | Up |
| mmu-miR-18a-5p | Up |
| mmu-miR-20a-5p | Up |
| mmu-miR-21a-5p | Up |
| mmu-miR-22-3p | Up |
| mmu-miR-23a-3p | Up |
| mmu-miR-29a-3p | Up |
| mmu-miR-29c-3p | Up |
| mmu-miR-27a-3p | Up |
| mmu-miR-31-5p | Up |
| mmu-miR-92a-3p | Up |
| mmu-miR-93-5p | Up |
| mmu-miR-96-5p | Up |
| mmu-miR-34a-5p | Up |
| mmu-miR-98-5p | Up |
| mmu-miR-103-3p | Up |
| mmu-miR-322-5p | Up |
| mmu-miR-148b-3p | Up |
| mmu-miR-345-5p | Up |
| mmu-miR-107-3p | Up |
| mmu-miR-10a-5p | Up |
| mmu-miR-17-5p | Up |
| mmu-miR-17-3p | Up |
| mmu-miR-19a-3p | Up |
| mmu-miR-25-3p | Up |
| mmu-miR-100-5p | Up |
| mmu-miR-139-5p | Up |
| mmu-miR-210-3p | Up |
| mmu-miR-214-3p | Up |
| mmu-miR-219a-5p | Up |
| mmu-miR-221-3p | Up |
| mmu-miR-222-3p | Up |
| mmu-miR-181c-5p | Up |
| mmu-miR-365-3p | Up |
| mmu-miR-483-3p | Down |
| mmu-miR-378a-3p | Up |
| mmu-miR-546 | Down |
| mmu-miR-494-3p | Up |
| mmu-miR-20b-5p | Up |
| mmu-miR-291b-5p | Down |
| mmu-miR-677-5p | Up |
| mmu-miR-497a-5p | Up |
| mmu-miR-423-3p | Down |
| mmu-miR-680 | Down |
| mmu-miR-682 | Up |
| mmu-miR-687 | Up |
| mmu-miR-690 | Up |
| mmu-miR-146b-5p | Up |
| mmu-miR-669b-5p | Down |
| mmu-miR-696 | Down |
| mmu-miR-491-5p | Down |
| mmu-miR-703 | Up |
| mmu-miR-705 | Down |
| mmu-miR-710 | Down |
| mmu-miR-711 | Down |
| mmu-miR-500-3p | Up |
| mmu-miR-718 | Down |
| mmu-miR-721 | Down |
| mmu-miR-652-3p | Up |
| mmu-miR-671-5p | Down |
| mmu-miR-672-5p | Down |
| mmu-miR-674-3p | Up |
| mmu-miR-712-3p | Down |
| mmu-miR-125a-3p | Down |
| mmu-miR-125b-2-3p | Up |
| mmu-miR-151-5p | Up |
| mmu-miR-186-3p | Down |
| mmu-miR-200b-5p | Up |
| mmu-miR-294-5p | Down |
| mmu-miR-300-5p | Down |
| mmu-miR-34c-3p | Down |
| mmu-miR-30c-1-3p | Down |
| mmu-miR-20a-3p | Up |
| mmu-miR-22-5p | Up |
| mmu-miR-31-3p | Up |
| mmu-miR-339-3p | Down |
| mmu-miR-139-3p | Down |
| mmu-miR-138-1-3p | Down |
| mmu-miR-466a-5p | Down |
| mmu-miR-483-5p | Down |
| mmu-miR-708-5p | Down |
| mmu-miR-193b-3p | Up |
| mmu-miR-877-5p | Down |
| mmu-miR-297c-5p | Down |
| mmu-miR-466c-5p | Down |
| mmu-miR-466f-5p | Down |
| mmu-miR-466h-5p | Down |
| mmu-miR-467c-5p | Down |
| mmu-miR-30c-2-3p | Down |
| mmu-miR-1224-5p | Up |
| mmu-miR-466j | Down |
| mmu-miR-669e-5p | Down |
| mmu-miR-1198-5p | Up |
| mmu-miR-1897-5p | Down |
| mmu-miR-1897-3p | Down |
| mmu-miR-1892 | Down |
| mmu-miR-1906 | Down |
| mmu-miR-1904 | Down |
| mmu-miR-1929-5p | Up |
| mmu-miR-1931 | Up |
| mmu-miR-1949 | Up |
| mmu-miR-669o-5p | Down |
| mmu-miR-1957a | Up |
| mmu-miR-1962 | Up |
| mmu-miR-1966-5p | Down |
| mmu-miR-1969 | Up |
| mmu-miR-1839-5p | Up |
| mmu-miR-1839-3p | Up |
| mmu-miR-1982-5p | Down |
| mmu-miR-1249-3p | Down |
| mmu-miR-2137 | Down |
| mmu-miR-432 | Up |
| mmu-miR-2861 | Down |
| mmu-miR-1843a-5p | Up |
| mmu-miR-3067-3p | Down |
| mmu-miR-3074-1-3p | Down |
| mmu-miR-3077-5p | Down |
| mmu-miR-3081-5p | Down |
| mmu-miR-3085-5p | Down |
| mmu-miR-3085-3p | Down |
| mmu-miR-466m-5p | Down |
| mmu-miR-466p-5p | Down |
| mmu-miR-3090-5p | Down |
| mmu-miR-3091-5p | Down |
| mmu-miR-3092-3p | Down |
| mmu-miR-3095-3p | Down |
| mmu-miR-3098-5p | Down |
| mmu-miR-3102-5p.2-5p | Down |
| mmu-miR-3473a | Up |
| mmu-miR-149-3p | Down |
| mmu-miR-187-5p | Down |
| mmu-miR-208a-5p | Down |
| mmu-miR-23a-5p | Up |
| mmu-miR-328-5p | Down |
| mmu-miR-350-5p | Down |
| mmu-miR-32-3p | Down |
| mmu-miR-211-3p | Down |
| mmu-miR-128-2-5p | Up |
| mmu-miR-760-5p | Down |
| mmu-miR-598-5p | Down |
| mmu-miR-669k-5p | Down |
| mmu-miR-669f-5p | Down |
| mmu-miR-1955-3p | Up |
| mmu-miR-664-5p | Up |
| mmu-miR-3960 | Down |
| mmu-miR-3963 | Up |
| mmu-miR-101c | Up |
| mmu-miR-3968 | Up |
| mmu-miR-3473b | Up |
| mmu-miR-5100 | Up |
| mmu-miR-5103 | Down |
| mmu-miR-5107-5p | Up |
| mmu-miR-5110 | Up |
| mmu-miR-5112 | Down |
| mmu-miR-5113 | Down |
| mmu-miR-5121 | Up |
| mmu-miR-5122 | Down |
| mmu-miR-5126 | Down |
| mmu-miR-5128 | Down |
| mmu-miR-5131 | Down |
| mmu-miR-5132-5p | Up |
| mmu-miR-5135 | Down |
| mmu-miR-5620-3p | Down |
| mmu-miR-5621-5p | Down |
| mmu-miR-5622-3p | Up |
| mmu-miR-344i | Down |
| mmu-miR-1929-3p | Up |
| mmu-miR-6238 | Up |
| mmu-miR-6240 | Down |
| mmu-miR-6347 | Down |
| mmu-miR-6348 | Down |
| mmu-miR-6349 | Down |
| mmu-miR-6360 | Up |
| mmu-miR-6368 | Up |
| mmu-miR-6370 | Up |
| mmu-miR-6378 | Up |
| mmu-miR-6388 | Down |
| mmu-miR-6392-3p | Down |
| mmu-miR-6394 | Down |
| mmu-miR-1957b | Up |
| mmu-miR-6399 | Down |
| mmu-miR-6402 | Up |
| mmu-miR-6415 | Down |
| mmu-miR-6418-5p | Down |
| mmu-miR-6538 | Down |
| mmu-miR-3473e | Down |
| mmu-miR-3475-5p | Down |
| mmu-miR-6906-5p | Up |
| mmu-miR-6907-5p | Down |
| mmu-miR-6908-5p | Down |
| mmu-miR-6909-5p | Up |
| mmu-miR-6912-5p | Up |
| mmu-miR-6922-5p | Down |
| mmu-miR-6923-5p | Up |
| mmu-miR-6926-5p | Up |
| mmu-miR-6929-3p | Down |
| mmu-miR-6931-5p | Down |
| mmu-miR-6937-5p | Up |
| mmu-miR-6937-3p | Down |
| mmu-miR-6940-3p | Up |
| mmu-miR-6942-5p | Down |
| mmu-miR-6946-5p | Down |
| mmu-miR-6948-5p | Up |
| mmu-miR-6949-5p | Up |
| mmu-miR-6950-5p | Up |
| mmu-miR-6954-5p | Down |
| mmu-miR-6955-5p | Up |
| mmu-miR-6961-5p | Down |
| mmu-miR-6963-5p | Up |
| mmu-miR-6965-5p | Up |
| mmu-miR-3547-5p | Down |
| mmu-miR-6972-5p | Up |
| mmu-miR-6978-5p | Down |
| mmu-miR-6982-5p | Down |
| mmu-miR-6984-5p | Down |
| mmu-miR-6987-5p | Up |
| mmu-miR-6995-5p | Down |
| mmu-miR-6998-5p | Up |
| mmu-miR-7001-5p | Up |
| mmu-miR-6973b-5p | Down |
| mmu-miR-7011-5p | Down |
| mmu-miR-7018-5p | Down |
| mmu-miR-7020-5p | Up |
| mmu-miR-7028-5p | Down |
| mmu-miR-7028-3p | Down |
| mmu-miR-7033-5p | Down |
| mmu-miR-7033-3p | Up |
| mmu-miR-7036a-5p | Up |
| mmu-miR-7040-5p | Down |
| mmu-miR-7042-5p | Up |
| mmu-miR-7044-5p | Down |
| mmu-miR-7045-5p | Down |
| mmu-miR-7045-3p | Down |
| mmu-miR-7046-5p | Down |
| mmu-miR-7047-5p | Up |
| mmu-miR-7048-5p | Down |
| mmu-miR-7049-5p | Down |
| mmu-miR-7050-5p | Up |
| mmu-miR-7052-5p | Down |
| mmu-miR-7058-5p | Up |
| mmu-miR-7066-5p | Down |
| mmu-miR-7082-5p | Down |
| mmu-miR-7088-5p | Down |
| mmu-miR-7089-5p | Down |
| mmu-miR-7115-3p | Down |
| mmu-miR-7118-5p | Down |
| mmu-miR-7218-5p | Down |
| mmu-miR-7218-3p | Down |
| mmu-miR-7221-5p | Down |
| mmu-miR-7222-3p | Down |
| mmu-miR-7226-5p | Down |
| mmu-miR-7235-5p | Down |
| mmu-miR-7238-5p | Down |
| mmu-miR-7241-3p | Down |
| mmu-miR-7648-5p | Down |
| mmu-miR-7648-3p | Down |
| mmu-miR-7036b-3p | Down |
| mmu-miR-292b-3p | Down |
| mmu-miR-3620-5p | Down |
| mmu-miR-3620-3p | Down |
| mmu-miR-290b-3p | Down |
| mmu-miR-7687-5p | Down |
| mmu-miR-8090 | Down |
| mmu-miR-8095 | Down |
| mmu-miR-8101 | Down |
| mmu-miR-8102 | Down |
| mmu-miR-8107 | Down |
| mmu-miR-8108 | Down |
| mmu-miR-8109 | Down |
| mmu-miR-8110 | Down |
| mmu-miR-8117 | Up |
| mmu-miR-3154 | Down |
| mmu-miR-9769-5p | Down |
