# Supplementary material for: Exosomes derived from bladder epithelial cells infected with uropathogenic Escherichia coli increase the severity of urinary tract infections (UTIs) by impairing macrophage function
Source: PLoS Pathog. 2024 Jan 8;20(1):e1011926. doi: 10.1371/journal.ppat.1011926 (PMC10798623; doi:10.1371/journal.ppat.1011926)
Supplement: S2 Table — (DOCX) [file ppat.1011926.s009.docx]

**S2 Table. Information for human subjects**

| **ID** | **Age(y)** | | **Gender** | **Diagnosis** | **Urine culture**  ***Escherichia coli* positive** | **Antibiotic therapy**  **for any reason** |
| --- | --- | --- | --- | --- | --- | --- |
| UTI-1 | | 53 | Male | Acute cystitis | Yes | No |
| UTI-2 | | 62 | Female | Acute cystitis | Yes | No |
| UTI-3 | | 31 | Female | Acute cystitis | Yes | No |
| UTI-4 | | 58 | Female | Acute cystitis | Yes | No |
| UTI-5 | | 76 | Male | Acute cystitis | Yes | No |
| UTI-6 | | 50 | Female | Acute cystitis | Yes | No |
| UTI-7 | | 43 | Female | Acute cystitis | Yes | No |
| UTI-8 | | 49 | Male | Acute cystitis | Yes | No |
| UTI-9 | | 65 | Male | Acute cystitis | Yes | No |
| UTI-10 | | 24 | Female | Acute cystitis | Yes | No |
| NC-1 | | 74 | Male | Health | No | No |
| NC-2 | | 40 | Male | Health | No | No |
| NC-3 | | 51 | Female | Health | No | No |
| NC-4 | | 26 | Female | Health | No | No |
| NC-5 | | 58 | Female | Health | No | No |
| NC-6 | | 69 | Male | Health | No | No |
| NC-7 | | 75 | Female | Health | No | No |
| NC-8 | | 31 | Male | Health | No | No |
| NC-9 | | 43 | Male | Health | No | No |
| NC-10 | | 36 | Female | Health | No | No |
