# Supplementary material for: Exosomes derived from bladder epithelial cells infected with uropathogenic Escherichia coli increase the severity of urinary tract infections (UTIs) by impairing macrophage function
Source: PLoS Pathog. 2024 Jan 8;20(1):e1011926. doi: 10.1371/journal.ppat.1011926 (PMC10798623; doi:10.1371/journal.ppat.1011926)
Supplement: S3 Table — (DOCX) [file ppat.1011926.s010.docx]

**S3 Table. Primers used for qRT-PCR**

| **Species** | **Gene name** | **Primer sequence（5ʹ‒3ʹ）** |
| --- | --- | --- |
| Mouse | *Tnfα* | F: GGTGCCTATGTCTCAGCCTCTT  R: GCCATAGAACTGATGAGAGGGAG |
| Mouse | *Actin* | F: GGTCCACACCCGCCACCAG  R: CACATGCCGGAGCCGTTGTC |
| Human | *TNFA* | F: CTCTTCTGCCTGCTGCACTTTG  R: ATGGGCTACAGGCTTGTCACTC |
| Human | hsa-miR-18a-5p | F: AGGCGCATTAAGGTGCATCTAGT  R: ATCCAGTGCAGGGTCCGAGG  RT:GTCGTATCCAGTGCAGGGTCCGAGGTATTCGCACTGGATACGACCTATCT |
| Human | *U6* | F: CTCGCTTCGGCAGCACA  R: AACGCTTCACGAATTTGCGT  RT: AACGCTTCACGAATTTGCGT |
