# Supplementary material for: Exosomes derived from bladder epithelial cells infected with uropathogenic Escherichia coli increase the severity of urinary tract infections (UTIs) by impairing macrophage function
Source: PLoS Pathog. 2024 Jan 8;20(1):e1011926. doi: 10.1371/journal.ppat.1011926 (PMC10798623; doi:10.1371/journal.ppat.1011926)
Supplement: S4 Table — (DOCX) [file ppat.1011926.s011.docx]

**S4 Table. Sequences of miRNA mimics**

| **Gene name** | **Sense（5'-3'）** | **Antisense（5'-3'）** |
| --- | --- | --- |
| mmu-miR-130a-3p | CAGUGCAAUGUUAAAAGGGCAU | GCCCUUUUAACAUUGCACUGUU |
| mmu-miR-21a-5p | UAGCUUAUCAGACUGAUGUUGA | AACAUCAGUCUGAUAAGCUAUU |
| mmu-miR-18a-5p | UAAGGUGCAUCUAGUGCAGAUAG | AUCUGCACUAGAUGCACCUUAUU |
| mimics N.C | UUGUACUACACAAAAGUACUG | GUACUUUUGUGUAGUACAAUU |
