# Supplementary material for: Exosomes derived from bladder epithelial cells infected with uropathogenic Escherichia coli increase the severity of urinary tract infections (UTIs) by impairing macrophage function
Source: PLoS Pathog. 2024 Jan 8;20(1):e1011926. doi: 10.1371/journal.ppat.1011926 (PMC10798623; doi:10.1371/journal.ppat.1011926)
Supplement: S5 Table — (DOCX) [file ppat.1011926.s012.docx]

**S5 Table. Antibody list**

| **Antibodies** | **Source** | **Identifier** |
| --- | --- | --- |
| [p-Erk1/2](https://scicrunch.org/resources/data/record/nif-0000-07730-1/AB_2315112/resolver?q=CAT%234370;&l=CAT%234370;&i=rrid:ab_2315112-2197276) Antibody used at 1:1000 | Cell Signaling Technology | Cat # 4370, *RRID*:AB_2315112 |
| Erk1/2 Antibody used at 1:1000 | Cell Signaling Technology | Cat # 4695, *RRID*:AB_390779 |
| p-SAPK/JNK Antibody used at 1:1000 | Cell Signaling Technology | Cat # 4668, *RRID*:AB_823588 |
| [SAPK/JNK](https://scicrunch.org/resources/data/record/nif-0000-07730-1/AB_2141027/resolver?q=CAT%239258&l=CAT%239258&i=rrid:ab_2141027-736750) Antibody used at 1:1000 | Cell Signaling Technology | Cat # 9258, *RRID*:AB_2141027 |
| p-p38 Antibody used at 1:1000 | Cell Signaling Technology | Cat # 4511, *RRID*:AB_2139682 |
| p38 Antibody used at 1:1000 | Cell Signaling Technology | Cat # 8690, *RRID*:AB_10999090 |
| p-p65 Antibody used at 1:1000 | Cell Signaling Technology | Cat # 3033, *RRID*:AB_331284 |
| p65 Antibody used at 1:1000 | Cell Signaling Technology | Cat # 8242, *RRID*:AB_10859369 |
| HSP90 Antibody used at 1:1000 | Cell Signaling Technology | Cat # 4877, *RRID*:AB_2233307 |
| CD63 Antibody used at 1:200 | Santa Cruz Biotechnology | Cat # sc-5275, *RRID*:AB_627877 |
| PTEN Antibody used at 1:1000 | Cell Signaling Technology | Cat # 9552, *RRID*:AB_10694066 |
| ACTB Antibody used at 1:20000 | ABclonal | Cat # AC026, RRID: AB_2768234 |
| HRP goat anti-rabbit IgG used at 1:3000 | ABclonal | Cat # AS014, RRID: AB_2769854 |
| HRP Goat Anti-Mouse IgG used at 1:3000 | ABclonal | Cat # AS003, RRID: AB_2769851 |
| Anti-mouse CD45 Alexa Fluor 700 used at 1:100 | Biolegend | Cat # 103128, RRID: AB_493715 |
| Anti-mouse F4/80 BV 605 used at 1:100 | Biolegend | Cat # 123133, RRID: AB_2562305 |
| Anti-mouse/human CD11b APC used at 1:100 | Biolegend | Cat # 101212, RRID: AB_312795 |
| Anti-mouse Ly-6G PE used at 1:100 | Biolegend | Cat # 127608; *RRID*:AB_1186099 |
| DAPI used at 1:100 | Boster | Cat # AR1176 |
| F4/80 (D4C8V) XP Antibody used at 1:200 | Cell Signaling Technology | Cat # 30325, *RRID*: AB_2798990 |
| Donkey anti-Mouse IgG (H+L) Secondary Antibody Alexa Fluor™ 555 used at 1:400 | Thermo Fisher | Cat # A-31570, *RRID*:AB_2536180 |
